# Genetic variants specific to aging-related verbal memory: Insights from GWASs in a population-based cohort

**DOI:** 10.1371/journal.pone.0182448

**Published:** 2017-08-11

**Authors:** Thalida E. Arpawong, Neil Pendleton, Krisztina Mekli, John J. McArdle, Margaret Gatz, Chris Armoskus, James A. Knowles, Carol A. Prescott

**Affiliations:** 1 Department of Psychology, Dornsife College of Letters, Arts and Sciences, University of Southern California, Los Angeles, California, United States of America; 2 Division of Neuroscience and Experimental Psychology, School of Biological Sciences, The University of Manchester, Manchester, United Kingdom; 3 Cathie Marsh Institute for Social Research, The University of Manchester, Manchester, United Kingdom; 4 Davis School of Gerontology, University of Southern California, Los Angeles, California, United States of America; 5 Department of Preventive Medicine, Keck School of Medicine, University of Southern California, Los Angeles, California, United States of America; 6 Department of Psychiatry and the Behavioral Sciences, Keck School of Medicine, University of Southern California, Los Angeles, California, United States of America; Nathan S Kline Institute, UNITED STATES

## Abstract

Verbal memory is typically studied using immediate recall (IR) and delayed recall (DR) scores, although DR is dependent on IR capability. Separating these components may be useful for deciphering the genetic variation in age-related memory abilities. This study was conducted to (a) construct individual trajectories in IR and independent aspects of delayed recall, or residualized-DR (rDR), across older adulthood; and (b) identify genetic markers that contribute to four estimated phenotypes: IR and rDR levels and changes after age 60. A cognitively intact sample (N = 20,650 with 125,164 observations) was drawn from the U.S. Health and Retirement Study, a nationally representative study of adults aged 50 and older. Mixed effects regression models were constructed using repeated measures from data collected every two years (1996–2012) to estimate level at age 60 and change in memory post-60 in IR and rDR. Genome-wide association scans (GWAS) were conducted in the genotypic subsample (N = 7,486) using ~1.2 million single nucleotide polymorphisms (SNPs). One SNP (rs2075650) in *TOMM40* associated with rDR level at the genome-wide level (p = 5.0x10^-08^), an effect that replicated in an independent sample from the English Longitudinal Study on Ageing (N = 6,898 with 41,328 observations). Meta-analysis of rDR level confirmed the association (p = 5.0x10^-11^) and identified two others in *TOMM40* (rs71352238 p = 1.0x10^-10^; rs157582 p = 7.0x10^-09^), and one in *APOE* (rs769449 p = 3.1 x10^-12^). Meta-analysis of IR change identified associations with three of the same SNPs in *TOMM40* (rs157582 p = 8.3x10^-10^; rs71352238 p = 1.9x10^-09^) and *APOE* (rs769449 p = 2.2x10^-08^). Conditional analyses indicate GWAS signals on rDR level were driven by APOE, whereas signals on IR change were driven by *TOMM40*. Additionally, we found that *TOMM40* had effects independent of *APOE* e4 on both phenotypes. Findings from this first U.S. population-based GWAS study conducted on both age-related immediate and delayed verbal memory merit continued examination in other samples and additional measures of verbal memory.

## Introduction

Memory impairment is the hallmark characteristic of mild cognitive impairment (MCI), Alzheimer disease (AD), and other types of dementia. It is estimated that currently (in 2016), ~5.2 million, or 11.1% of the 47 million persons aged 65 or older in the United States will develop AD in their lifetimes [1]. With an expanding population of older adults, these figures are expected to increase by 2050 to ~13.7 million, or 15.6% of the 87.7 million persons aged 65+ [1]. Verbal memory ability has particular relevance to aging research in that there is normative yet profound decline in older age [2–4]. The molecular basis of such decline is increasingly being disentangled as is the intricate relationship between decline and memory-related deficits [5–10]. Identifying the extent to which genetic variants underlie individual differences in specific aspects of memory trajectories are useful for understanding disease-related mechanisms that contribute to memory impairments as well as age-related memory changes.

### Distinguishing immediate and delayed recall

Verbal memory is one aspect of episodic memory that requires verbal processing for encoding or retrieval of words, sentences, or prose [11]. It is typically assessed using both immediate recall and delayed recall tasks, which represent distinct cognitive processes [12, 13]. Immediate recall involves use of working memory, by which recently-presented information is rehearsed until recollection. Delayed recall requires intact immediate recall as it assesses retrieval of the information learned during the initial processing involved in immediate recall. This conceptual distinction between immediate and delayed recall is supported by functional brain imaging studies suggesting different localization of these processes [14–18]. Moreover, each component has distinct clinical relevance and implications when studying the underlying genetics.

Studies involving candidate genes have found learning and retrieval are partially distinct. For example, a study of community-dwelling older adults (ages 59 to 95) evaluated change in cognitive performance, comparing individuals with the Apolipoprotein E (*APOE)* e3/e3 and e3/e4 genotypes to assess the effect of the e4 risk allele. With regard to verbal memory, e3/e4 individuals exhibited significantly greater decline in delayed recall but not immediate recall [19]. In another study among healthy older adults (ages 60 to 100), the catechol-O-methyltransferase (*COMT*) enzyme was evaluated for effects on prefrontal cognitive functions. While there were no associations found between *COMT* genotypes and verbal memory overall, upon testing differences in the association by gender, men who were homozygous for the Val allele (vs. Val/Met and Met/Met) performed better on delayed recall only [20]. There were no differences in either recall phase by genotype that were detected for women. These studies provide preliminary indication that there is a distinct molecular basis for immediate and delayed recall.

From the clinical perspective, mild impairments in recall of verbal information over time are commonly experienced in aging and not considered pathological, whereas greater impairments particularly in delayed recall is a key early sign of Alzheimer disease and other dementias. Delayed recall performance has been shown to be a better predictor than immediate recall of AD severity, conversion to AD in the dementia process, or in differentiating normative changes from MCI and AD [21–23]. Despite the clinical importance, decline in delayed recall independent of decline in immediate memory has not been well-characterized. Generally, longitudinal studies report steady decline in overall verbal memory performance begins around 60 years of age [2, 24–26]. Distinguishing patterns in decline for immediate and delayed verbal recall is critical because of the potential differences in age-related trajectories as well as differences in implications for disease identification and prognosis.

### Distinguishing memory level and change

A related issue for studying trajectories of memory over age is whether the factors underlying *level* of memory performance in early life and midlife differ from the processes that produce memory *decline* in later life. To use an analogy from physical growth, it seems unlikely that the (behavioral and genetic) factors contributing to loss of height in later life are the same as those contributing to stature in early adulthood. There has been increasing support of this age effect with episodic memory, and other cognitive aging phenotypes, as more studies have found that the effect of genes in older age (e.g., *APOE*, *KIBRA*, *BDNF*, *DRD2*, *GRM3*, *COMT*) tends to be stronger [27–30]. In parallel, evidence from longitudinal studies of aging twins indicates that inter-individual variation in level is highly influenced by genetic factors, but the genetic factors contributing to memory level are partially distinct from those underlying memory decline [28, 31–41]. Taken together, these suggest that distinguishing between level and change in verbal memory abilities would be useful for detecting underlying genetic relationships.

### Study aims

This study addresses three important limitations common in genetic research on memory decline in aging: (a) confounding of measurement of delayed memory with immediate memory and (b) reliance on candidate genes. We are unaware of any prior study that has used a genome-wide approach to identify unique genetic associations with immediate and delayed verbal recall.

The first aim of this study was to characterize trajectories of four discrete components of verbal memory across older adulthood: level and decline in immediate recall, and level and change in independent aspects of delayed recall. This research employs data from a large population representative sample (N = 20,650) of older individuals from the U.S. Health and Retirement Study (HRS), with repeated measures (up to eight assessments per person) of verbal memory.

Our second aim was to identify genetic variants that associate with individual differences in the four components and to evaluate whether the associations are specific to each component. We employed genome wide association scans (GWAS) that had broad coverage of ~1.2 million SNP markers with the HRS genetic subsample (N = 7,486), followed by GWAS in an independent replication sample from the English Longitudinal Study of Ageing (ELSA; N = 6,898).

## Material and methods

### Participants

Discovery cohort. The Health and Retirement Study (HRS) is a nationally representative longitudinal study of U.S. households containing adults aged 50 years and older, with interviews conducted every two years, starting in 1992. Analyses of memory trajectories are based on data from a phenotypic sample comprising HRS participants who had completed at least two assessments of verbal recall between 1996 and 2012 and were at least 50 years of age during the study. Scores for some individuals were excluded if recorded prior to their turning age 50. We excluded scores of individuals who had a likely diagnosis of dementia, based on a question to the respondent on whether they had been diagnosed by a healthcare provider (with Alzheimer disease, dementia, senility, or serious memory-related problem) or the respondent requiring a proxy to complete their interview. Memory scores for these individuals were excluded for the wave in which the diagnosis was reported, the wave prior to reporting the diagnosis, and all subsequent waves when the individual was interviewed. These criteria yielded a sample of N = 20,650, with individuals assessed up to 8 times over the 16 years (125,164 observations total), with an average of 6.1 assessments per person. This phenotypic sample is 57.9% female, and the self-reported racial/ethnic composition is 82.2% Non-Hispanic White (NHW), 14.1% African-American/Black, and 3.8% Hispanic/Latino.

To reduce potential bias from population stratification bias and increase comparability with the ELSA replication sample, the GWAS was limited to individuals who identified as NHW (i.e., having primarily European ancestry). Other selection criteria were having verbal memory data on at least two occasions and providing a DNA sample, resulting in N = 7,486 (57.6% female) with 65,937 observations.

Replication cohort. The English Longitudinal Study of Ageing (ELSA) is a companion study of HRS, with a nationally representative cohort of individuals living in England aged 50 and older. The study began in 2002 with 11,391 participants and has since included interviews every two years, with measures collected for the purposes of harmonizing with HRS, including assessment of verbal memory (see Steptoe et al., 2013 [42]). Inclusion criteria for the present study were the same as for the HRS discovery sample. Memory scores and genetic information were available for a sample of N = 6,898 unrelated individuals (53.8% female) assessed up to 6 times (41,328 observations, collected 2002–2012).

### Measures

#### Memory recall scores

In HRS and ELSA, a verbal memory task was administered as part of a series of cognitive tests, based on the Modified Telephone Interview for Cognitive Status (TICS-m [43]). This assessment was designed to screen for serious impairment and to aid in characterizing cognitive decline and mild impairment in large-scale community based studies of older adults where it is impractical to conduct more detailed assessments. This format is administered in many large-scale population-based studies conducted in the U.S. and internationally [42, 44–47].

In both HRS and ELSA, immediate recall (IR) and delayed recall (DR) were assessed as follows: Participants were alerted to pay attention and listen carefully, then asked to confirm readiness prior to being read a list of 10 nouns (administered by interviewer or taped recording) at the rate of one per second; then they were asked to recall the words immediately and again after a 5-minute delay [48] which was filled by asking non-cognitive survey items. The possible range of scores for each test is 0 to 10, for number of words recalled correctly, in any order. In HRS, the first assessment was typically in-person whereas most subsequent assessments were by telephone. For respondents age 80 or older, follow-ups were conducted face-to-face. Between 1996 and 2012, HRS used alternating lists across waves to reduce practice effects [44]. McArdle and colleagues studied construct equivalence of the cognitive measures in HRS and confirmed measurement invariance across waves of testing and identified only small effects from modality of testing [44, 49]. IR and DR scores were used as dependent variables as was a residual delayed recall (rDR) score. The rDR score represents the component of DR that is independent of IR (See pages 1–3 in S1 File).

#### Genetic marker data

For HRS, genotype data were accessed from the National Center for Biotechnology Information Genotypes and Phenotypes Database (dbGaP [50]). DNA samples from HRS participants were collected in two waves. In 2006, the first wave was collected from buccal swabs using the Qiagen Autopure method (Qiagen, Valencia, CA). In 2008, the second wave was collected using Oragene saliva kits and extraction method. Both waves were genotyped by the NIH Center for Inherited Disease Research (CIDR; Johns Hopkins University) using the HumanOmni2.5-4v1 array from Illumina (San Diego, CA). Raw data from both phases were clustered and called together. HRS followed standard quality control recommendations to exclude samples and markers that obtained questionable data, including CIDR technical filters [51], removing SNPs that were duplicates, had missing call rates ≥ 2%, > 4 discordant calls, > 1 Mendelian error, deviations from Hardy-Weinberg equilibrium (at *P*-value < 10^−4^ in European samples), and sex differences in allelic frequency ≥ 0.2). Further detail is provided in HRS documentation [52]. To reduce generating inflated P-values from inclusion of rare SNPs, we excluded SNPs with a MAF < 5%. Applying these criteria resulted in available data on 1,198,956 SNPs for the 9,532 NHW individuals, of whom 7,486 had phenotypic data and form our analytic sample.

For ELSA, genotype data were accessed from the European Genome-phenome Archive (EGA; http://www.elsa-project.ac.uk/). DNA was extracted from blood samples collected in 2004 and 2008, and samples were genotyped by University College London (UCL) Genomics Institute on the HumanOmni2.5–8 array from Illumina (San Diego, CA). Because the two studies were designed to be similar (with 2,368,902 markers in common across the studies), the same quality control procedures used with HRS were applied to the ELSA data, except that UCL excluded SNPs with missing call rates >5% (rather than 2%), yielding available data on 1,204,603 SNPs for 7,404 individuals with European ancestry, of whom 6,898 had phenotypic data also.

### Statistical analysis

As detailed below, we followed five analytical steps: (1) Phenotype construction. Mixed effects regression models were used to evaluate the age-based trajectories and between-subject variation in trajectories of IR and rDR (analyzed separately). Four phenotypes were estimated for each individual: IR level, IR change, rDR level, and rDR change. (2) GWAS. Separate GWASs were run, first in the discovery cohort, to identify genetic loci associated with each of the four phenotypes. Replication GWASs were run in the independent cohort to evaluate replication. (3) SNP Comparison. We compared GWAS results across the phenotypes, and between the discovery and replication cohorts. (4) Meta-analysis. We conducted a meta-analysis of the discovery and replication cohorts to assess the robustness of the combined effects. (5) Functional genomic and pathway analysis. We evaluated the plausible biological pathways in which implicated genes play a role in memory outcomes.

#### Phenotype construction

To isolate the component of delayed recall that is independent of immediate recall, we formed a residualized-delayed recall (rDR) score. Mixed effects regression models were used to regress DR on IR using data from all participants and observations. rDR is the residual value (difference between the observed DR score and predicted DR score based on IR). IR and rDR scores were used as dependent variables in spline modeling to obtain four estimates for each participant to be used as phenotypes in genetic analyses: immediate recall level (IR-L), immediate recall change (IR-C), residual delayed recall level (rDR-L), and residual delayed recall change (rDR-C).

Splines are piecewise segments that fit linear trends, but each spline has its own intercept and slope to reflect the changing trends of the score over age. We conducted preliminary analyses to identify the number of splines needed to summarize the overall trajectories in the data and to identify the knot points (the dividing point between splines). The two-spline model was selected as the most parsimonious for characterizing overall trajectories for both IR and rDR. Based on prior literature [2, 24–26] and preliminary analyses, age 60 was selected as the spline knot point, i.e., allowing individuals to have different slopes before and after age 60.

All spline models were conducted as mixed effects analyses using PROC MIXED in SAS 9.4 [53] to estimate subject-specific intercepts and linear slopes, using empirical Bayes methods [54]. This technique allowed us to create comparable scores for participants, regardless of their age and duration of participation (as long as they had two observations after age 60) and is similar to the approach used to estimate level and decline of other cognitive measures in HRS [55, 56].

#### Genome-wide association scans

Separate GWASs, run as linear regressions under an additive model, were conducted for each of the four phenotypes (IR-L, IR-C, rDR-L, and rDR-C), adjusting for sex and population stratification (the first three principal components). As with any statistical analysis of association, if the correlation between dependent and independent variables differs for subpopulations, spurious associations may result in GWAS [57]. To reduce such type 1 error, we conducted GWAS adjusting for population structure as indicated by latent factors from principal components analysis (PCA) [58, 59]. Detailed descriptions of the processes employed for running PCA, including SNP selection, are provided by HRS [52], and follow methods outlined by Patterson and colleagues [60]. Two PCAs were run. The first PCA included 1,230 HapMap anchors from various ancestries, and were used to test against self-reported race and ethnic classifications. Several corrections to the dataset were made based on this analysis. The second PCA was run on the corrected dataset, on unrelated individuals and excluding HapMap anchors, to create eigenvectors to serve as covariates and adjust for population stratification in association tests. From the second PCA, the first two eigenvalues with the highest values accounted for less than 4.5% of the overall genetic variance, with additional components (3–8) increasing this minimally, by a total of ~1.0% [52]. We conducted correlational analysis for IR and DR level and change scores and found the first eight components had small and non-significant associations (all *r*’s<0.02) with each of the four phenotypes. As a check for non-linear associations, we examined graphs in which level of immediate recall was divided into quartiles and plotted against the first three principal components (PCs) (Supporting Information, Fig A-C in S1 Fig). The pattern did not suggest population substructures consistent with false associations in GWAS. Based on all this, we opted for a strategy that does not ignore population substructure, but also does not over-correct, and adjusted for the first three PCs in all analyses. This is a similar approach used in another recent GWAS conducted using HRS data (e.g., Ramanan et al., 2014). When coupling this approach of adjusting for PCs with all quality control procedures performed, excluding any related individuals and limiting the dataset for ancestral homogeneity, we reduce the likelihood of false associations resulting from population stratification [59–65]. All GWASs were completed using PLINK 1.9 [66, 67].

To estimate the fraction of phenotypic variation explained by common SNPs (with minor allele frequency ≥ 5%) for each phenotype, we implemented genomic-relationship-matrix restricted maximum likelihood (GREML) in unrelated individuals from all genotyped SNPs in the data set. We included only individuals falling within 1 SD of all self-identified non-Hispanic Whites for eigenvectors 1 and 2 in the principal components analysis of all unrelated participants. More information on the process for screening and selection of the sample by ancestral homogeneity and relatedness is provided in HRS documentation [52]. SNP heritability estimates were run adjusted for sex and three PCs. GREML was implemented in the software Genome-Wide Complex Trait Analysis (GCTA [68]) version 1.26.0.

#### SNP comparison

We evaluated SNP associations in the GWASs by p-value, using the genome-wide significance level of *p* ≤ 5.0x10^-08^ [69]. We also discuss additional findings at the “suggestive association” level of *p* ≤ 5.0x10^-06^ because we prefer not to miss potential new findings (Type II error) at the cost of an overly stringent Type I cutoff. To compare SNP associations from discovery and replication data sets we: 1) assessed the independent contribution of SNPs meeting genome-wide significance in the discovery scan; and 2) compared effect sizes and p-values to those estimated for these SNPs in the replication scans. For visualization of the SNP plots, we used R (CRAN; https://www.r-project.org) and LocusZoom v1.1 [70].

#### Meta-analysis

We conducted a meta-analysis of the discovery and replication cohorts using the inverse variance weighted model approach implemented in the METAL package (http://www.sph.umich.edu/csg/abecasis/Metal [71]).

### Functional genomic and pathway analysis

Genes and flanking genes were identified using dbSNP and the “SNP and CNV Annotation Database” (SCAN) (http://www.scandb.org/newinterface/index.html). Gene functions were identified using the literature search feature of the “Gene Expression Analysis” (GEA) tool (http://chrisarm.com/gea/lsearch/). Gene network analysis was conducted using the Ingenuity Knowledge Base and Ingenuity Pathway Analysis (IPA, Ingenuity Systems, Redwood City, CA; http://www.qiagenbioinformatics.com/products/ingenuity-pathway-analysis/). Additional interactions were added sourced from the Qinsight Biomedical Search Engine (Quertle LLC; Henderson, NV; https://www.quetzal-search.info/) and the BaseSpace Correlation Engine (Illumina, San Diego, CA; https://basespace.illumina.com/home/index), which summarizes evidence from the National Center for Biotechnology Information (NCBI) Gene Expression Omnibus (GEO; https://www.ncbi.nlm.nih.gov/geo/).

## Results

### Sample characteristics and outcome variables

Table 1 provides selected characteristics for HRS participants in the full phenotypic sample (N = 20,650) and the NHW genotypic sample used for the GWAS (N = 7,486). The mean IR and DR scores are based on the first assessment occasion for each individual. In the phenotypic sample, the mean score (out of 10 words) was 5.7 for IR and 4.5 for DR. The full HRS sample and the NHW phenotypic sub-sample are similar in score distributions and gender ratio, but the genotypic sample was slightly younger and had memory scores that averaged 0.2 SD for both IR and DR. After adjusting for a linear effect of age, GWAS sample membership was not associated with IR or DR scores (*ß* = 0.02).

**Table 1:** Characteristics of HRS participants at the first assessment point, tested for immediate and delayed recall between 1996 and 2012, in the HRS full and Non-Hispanic White (NHW) genotypic samples.

	Full HRS Sample (N = 20,650)		NHW Genotypic Sample (N = 7,486)	
	Mean (SD)	%	Mean (SD)	%
Age (range 50–103)	64.4 (9.8)		61.7 (8.2)	—
Female	—	57.9	—	57.9
Male	—	42.1	—	42.1
Race/Ethnicity				
Non-Hispanic white	—	82.2	—	100.0
Hispanic/Latino	—	14.1	—	—
African-American	—	3.8	—	—
Immediate recall score^a^	5.7 (1.8)	—	6.1 (1.6)	—
Delayed recall score^a^	4.5 (2.2)	—	5.0 (2.0)	—

^a^ Immediate and delayed recall scores ranged from 0 to 10.

**Fig 1:**
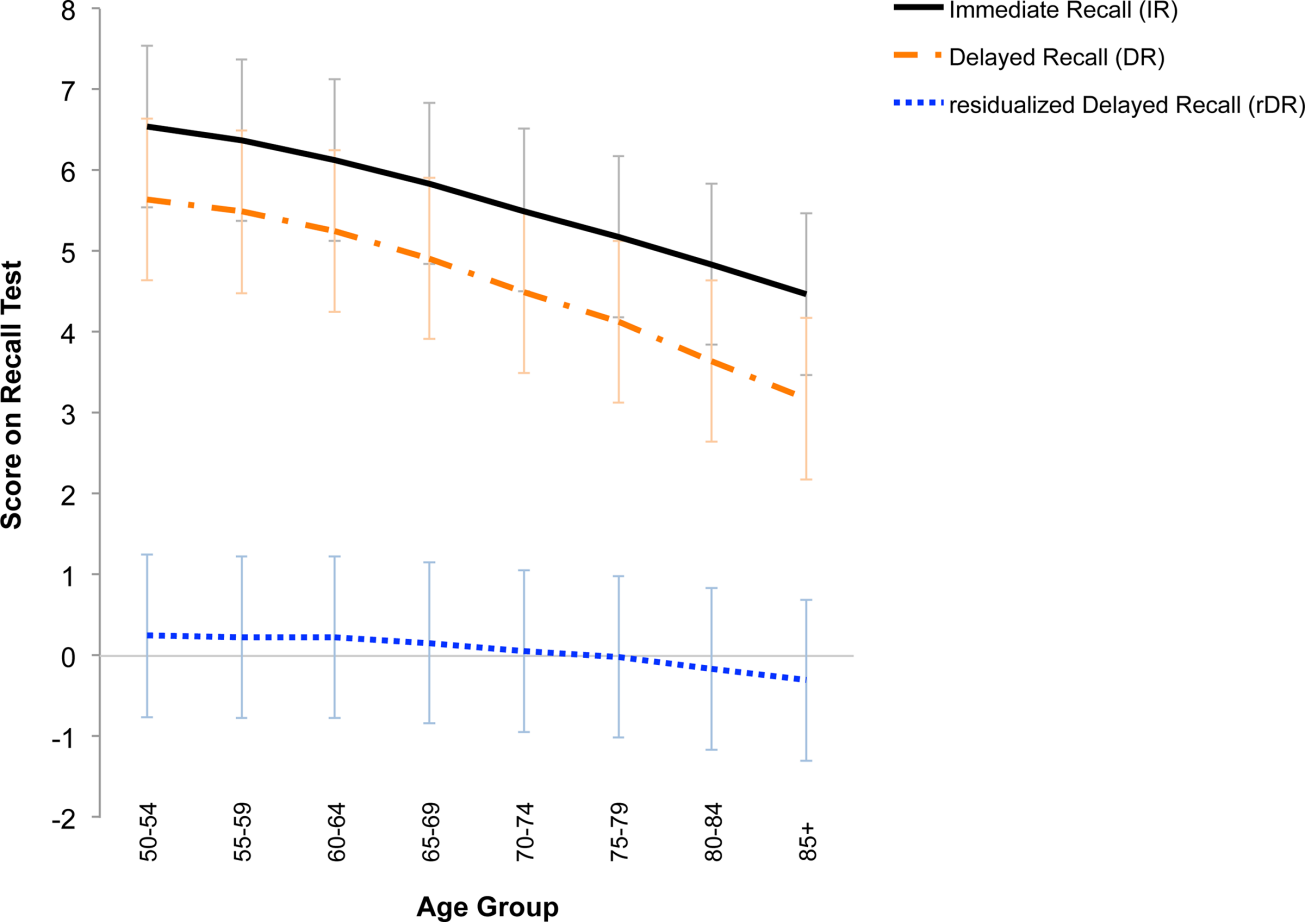
Scores (as words recalled) for immediate, delayed, and residualized delayed recall among 20,650 participants in the HRS.

In the full phenotypic HRS sample, IR was strongly correlated with DR (r = .77), but (as expected) not with residualized delayed recall (rDR; r = -.01), reflecting our goal of isolating the immediate and delayed components of verbal recall. Fig 1 shows recall scores, including the overlap of IR and DR, as well as rDR, or the independent component of delayed scores after partialling out immediate scores.

Aim 1 of the study was to characterize the longitudinal trajectories of the phenotypes: IR level (IR-L), IR change (IR-C), residual DR level (rDR-L) and residual DR change (rDR-C). To achieve this, we calculated and compared the estimated parameters from spline models for the four phenotypes for the HRS full phenotypic sample. The first slope component (s1) is a linear function of the scores obtained prior to age 60 and a second slope component (s2) is a linear function of scores obtained after age 60. There turned out to be negligible variation in slope prior to age 60 (i.e., memory performance was stable for the majority of participants; IR-C variance = 0.028, DR-C variance = 0.006, rDR-C = 0.004). Because there was more between-person variation after age 60 for IR and DR (IR-C variance = 0.094, DR-C variance = 0.113, rDR-C = 0.004), the key model parameters are subject-specific intercepts (random effects) for memory level at age 60 (i.e., representing IR-L or rDR-L) and subject-specific estimates of slope (change) after age 60, representing IR-C or rDR-C. However, variance in rDR-C post-60 was considerably smaller than for IR-C and DR-C.

As shown in Table 2, higher recall level negatively correlated with decline for both IR (*r* = -.62) and DR (*r* = -.64), indicating that individuals with initially better recall had more rapid decline, whereas those who began lower did not decline as much across subsequent measurements. In contrast, rDR level was only weakly associated with decline (*r* = -.18), suggesting that change on recall is independent of ability.

**Table 2:** Age-adjusted values for estimated immediate recall (IR), delayed recall (DR), and residual delayed recall (rDR) in HRS.

	Genotypic Sample (N = 7,486)						
	Mean	SD	1	2	3	4	5
1. IR level	6.17	0.89					
2. IR change	-0.50	0.30	-0.62				
3. DR level	5.23	1.11	0.85	-0.49			
4. DR change	-0.67	0.36	-0.55	0.76	-0.64		
5. rDR level	0.22	0.34	0.15	-0.08	0.62	-0.31	
6. rDR change	-0.20	0.10	-0.03	-0.01	-0.19	0.55	-0.18

SD = standard deviation. Immediate and delayed recall scores represent the number of words recalled correctly, between 0 and 10. Correlations of .03 or greater had p-values < 0.01. rDR scores are IR-adjusted. IR and rDR level are estimated scores for age 60. IR and rDR change are estimated linear slopes per decade after age 60.

### Evaluation of GWAS results

Quantile-quantile (QQ) plots for the HRS sample were constructed to show the ratio of observed to expected p-values for each SNP in the four GWASs, each adjusted for three PCs (see Figs A-D in S2 Fig). The majority of SNPs fell within the 95% confidence interval of the expectation; thus, it is reasonable to assume that there is little concern for inflation (type 1 error) of the observed p-values. The genomic inflation factor (lambda) for each scan was close to 1 (range λ’s = 1.001 to 1.010), and thus, not suggestive of problems with population stratification [72]. Notably, comparison scans using eight PCs showed nearly identical effect sizes and a similar pattern of p-values, and hence the three PCs controlled for in the analyses appear sufficient. Similarly, QQ plots for the ELSA sample did not indicate problems with population stratification (see Figs A-D in S3 Fig).

#### GWAS findings

**Fig 2:**
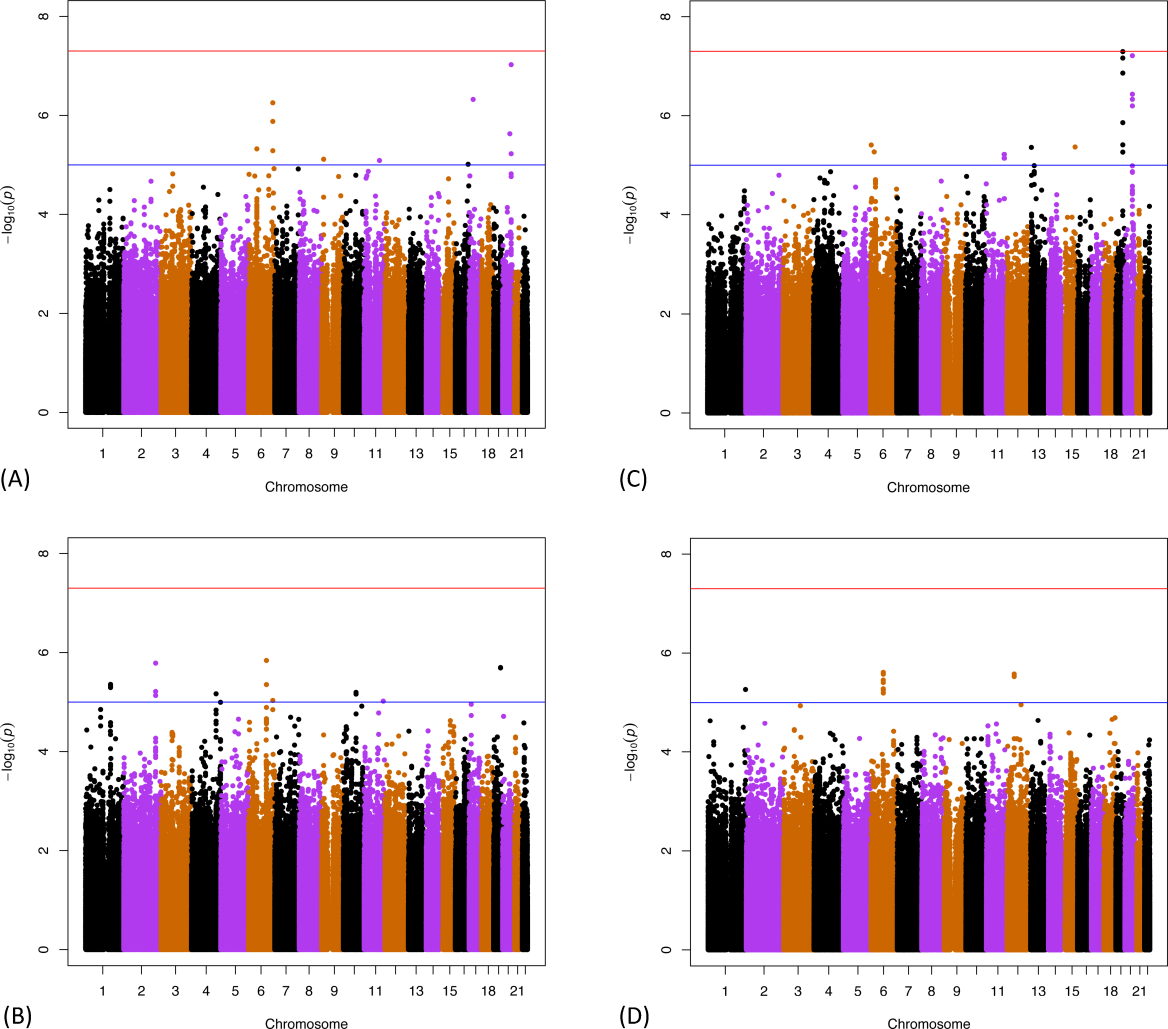
P-values for all 1,198,956 SNP associations from GWAS on the HRS genetic sample. (A) Level of Immediate Recall (IR-L); (B) Change in Immediate Recall (IR-C); (C) Level of Residualized Delayed Recall (rDR-L); (D) Change in Residualized Delayed Recall (rDR-C). For these figures, the upper (red) horizontal line demarcates the threshold of p = -log(5.0x10^-08^) and the lower (blue) horizontal line demarcates p = -log(1x10^-05^). SNPs are arranged by their chromosomal position (x-axis).

Fig 2 shows Manhattan plots for the discovery GWAS in HRS, for IR-L, IR-C, rDR-L, and rDR-C, respectively. The x-axis shows each of the 1.2M SNPs arranged in order of chromosomes and map location. The y-axis shows the log of the p-value for the association between the SNP and the phenotype. One SNP in the scan for rDR-L (Fig 2C) surpassed the genome-wide significance threshold (*p* ≤ 5.0x10^-08^), and several other SNPs in all four scans met our criterion for suggestive association (*p* ≤ 5.0x10^-06^). Corresponding Manhattan plots for the ELSA sample are provided in the Supporting Information (Figs A-D in S4 Fig).

Overall, there were 29 SNPs that exceeded the *p*≤5.0x10^-06^ cutoff for a suggestive association with one or more phenotypes. These are listed in Table 3, ordered by chromosome, and chromosomal location, and presented with the associated phenotype, corresponding gene location, minor allele, effect size, and p-value from the HRS GWAS. Also shown are the effect sizes and p-values from the ELSA association tests. Additional findings found in the ELSA cohort only are provided in Supporting Information (Table C in S1 File).

**Table 3:** Strongest associated SNPs detected by GWASs in the HRS discovery sample and comparisons in the ELSA replication sample, for immediate recall level (IR-L) and change (IR-C) and residual delayed recall level (rDR-L) and change (rDR-C).

Pheno.	Chr.	SNP	Position	Ref. allele	Minor Allele	Gene(s)	HRS		ELSA	
					(freq.)		Beta	P	Beta	P
IR-C	1	rs11804244	163212633	G	G (0.150)	*LOC100113374* or *NUF2*	-0.029	4.E-06	0.004	1.E-01
IR-C	1	rs6704030	163218278	G	C (0.149)	*LOC100113374* or *NUF2*	-0.029	5.E-06	0.004	2.E-01
IR-C	2	rs12463410	207715608	A	T (0.021)	*FASTKD2* ^a^ or *CP*O ^a^	-0.054	2.E-06	0.001	8.E-01
rDR-L	6	rs9504162	4599442	A	T (0.187)	*LOC100129052* or *KU-MEL-3*	-0.028	4.E-06	0.002	8.E-02
IR-L	6	rs7766458	54140128	C	C (0.335)	*C6orf142* or *TINAG*	-0.095	5.E-06	0.003	9.E-01
rDR-C	6	rs12195716	79535412	G	T (0.497)	*LOC100128162* or *IRAK1BP1*	-0.005	4.E-06	0.000	9.E-01
rDR-C	6	rs2174743	79591805	A	G (0.488)	*IRAK1BP1*	-0.005	4.E-06	0.000	9.E-01
rDR-C	6	rs7756858	79619968	G	A (0.460)	*IRAK1BP1* or *PHIP* ^a^	-0.005	2.E-06	0.000	9.E-01
rDR-C	6	rs1572585	79690576	A	C (0.494)	*PHIP* ^a^	-0.005	3.E-06	0.000	1.E+00
IR-C	6	rs9372456	116592956	G	G (0.384)	*RPS5P1* or *TSPYL1*	0.028	1.E-06	-0.003	4.E-01
IR-C	6	rs10581090	116658967	A	C (0.482)	*DSE* ^a^	0.026	4.E-06	-0.004	2.E-01
IR-L	6	rs147391086	157984507	A	A (0.015)	—	-0.153	1.E-06	0.038	2.E-01
IR-L	6	rs150510877	157994175	A	T (0.019)	—	-0.159	6.E-07	0.032	2.E-01
rDR-C	12	rs10880835	46079245	C	G (0.498)	*LOC100131290* or *LOC400027*	0.005	3.E-06	na	na
rDR-C	12	rs35162469	46081065	A	A (0.485)	*LOC100131290* or *LOC400027*	0.005	3.E-06	0.000	8.E-01
rDR-L	13	rs9510784	24188270	A	C (0.450)	*TNFRSF19* ^a^	-0.025	4.E-06	0.005	5.E-01
rDR-L	15	rs4076414	86441452	C	G (0.418)	*KLHL25* or *AGBL1*	0.025	4.E-06	0.013	9.E-02
IR-L	17	rs8072199	26116848	A	T (0.227)	*NOS2A*	0.074	5.E-07	-0.002	9.E-01
IR-C	19	rs283815	45390333	G	G (0.304)	*PVRL2* ^a^	-0.032	2.E-06	-0.012	2.E-04
rDR-L							-0.031	4.E-06	-0.027	3.E-03
rDR-L	19	rs71352238	45394336	G	C (0.085)	—	-0.043	7.E-08	-0.039	2.E-04
rDR-L	19	rs2075650	45395619	G	G (0.119)	*TOMM40* ^a^	-0.043^b^	5.E-08	-0.041	1.E-04
IR-C	19	rs157582	45396219	A	T (0.294)	*TOMM40* ^a^	-0.032	2.E-06	-0.013	9.E-05
rDR-L							-0.033	1.E-06	-0.030	9.E-04
rDR-L	19	rs769449	45410002	A	A (0.065)	*APOE* ^a^	-0.045	1.E-07	-0.053	5.E-06
IR-L	20	rs11906369	46829233	A	A (0.146)	*LOC100130372* or *LOC284749*	-0.123	2.E-06	na	na
rDR-L	20	rs6512614	48792663	C	C (0.483)	*TMEM189* ^a^ or *CEBPB* ^a^	-0.028	5.E-07	-0.005	5.E-01
rDR-L	20	rs6012871	48792992	G	C (0.486)	*TMEM189* ^a^ or *CEBPB* ^a^	-0.028	6.E-07	-0.006	4.E-01
rDR-L	20	rs6125931	48795413	G	G (0.484)	*TMEM189* ^a^ or *CEBPB* ^a^	-0.028	4.E-07	-0.004	6.E-01
rDR-L	20	rs6125934	48803937	A	T (0.396)	*TMEM189* ^a^ or *CEBPB* ^a^	-0.030	6.E-08	-0.004	6.E-01
IR-L	20	rs6025261	55503259	A	A (0.286)	*PTMAP6* or *LOC728902*	0.079	9.E-08	0.008	5.E-01

Genome build GRCh37. Pheno = phenotype; Chr = chromosome; Ref. allele = reference allele; freq = frequency.—Indicates that a gene name in which the SNP resides has not been identified. na = not available because when quality control filters were applied to the genotyped data, some SNPs were excluded from the analyses with ELSA. SNPs in the table are those with associations of p≤5x10-06 in the discovery cohort, HRS. When two gene names are provided, they represent the closest left and right flanking genes, respectively.

^a^ Indicates there are prior reported associations with the gene and memory phenotypes (source: PubMed).

^b^ Indicates the SNP association surpassed the genome-wide significance threshold of 5E-08 in the HRS discovery cohort and replicated in the independent cohort at p < .05.

**Fig 3:**
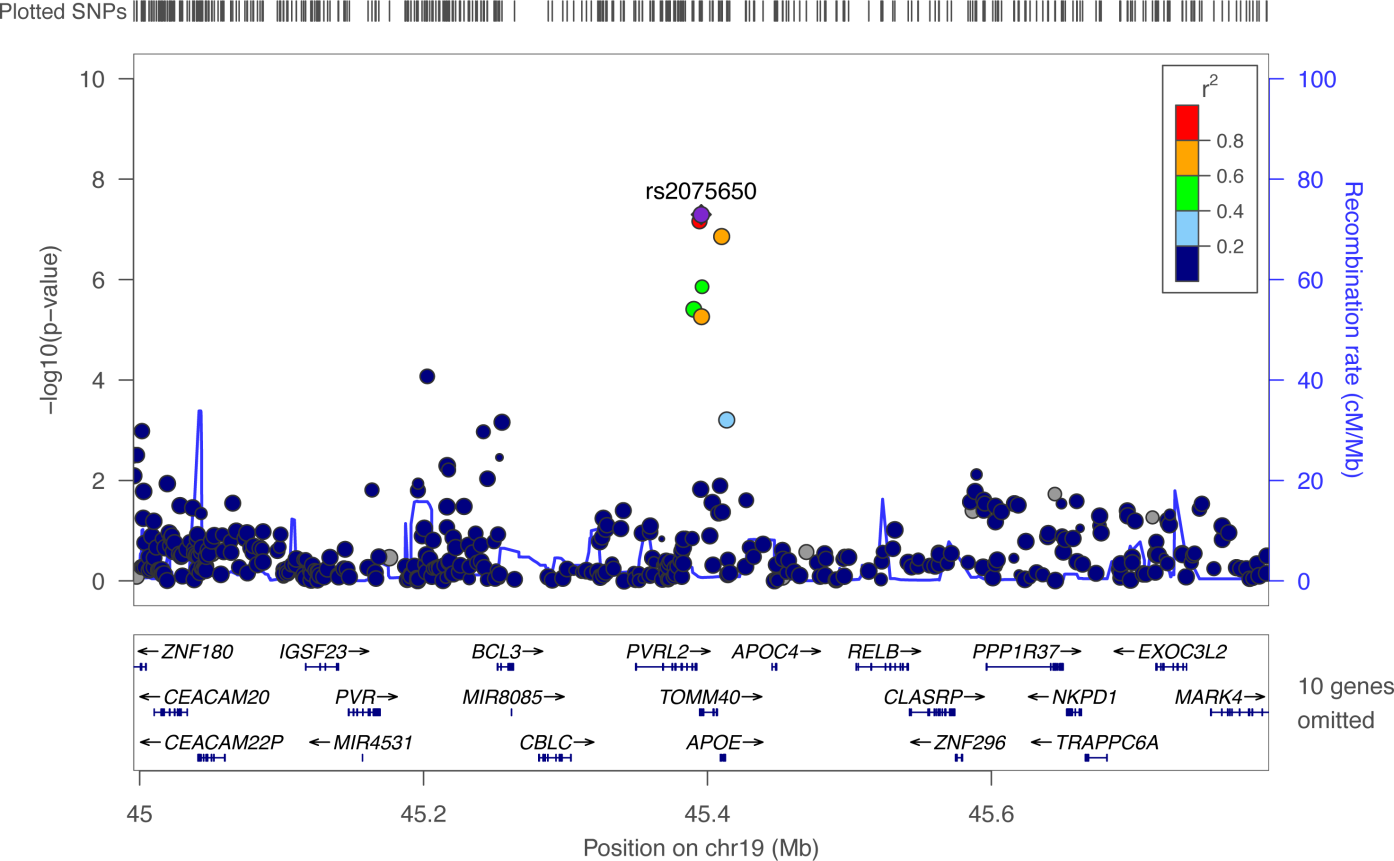
Regional plot showing the results for the association between rDR-L in HRS, with the top SNP in *TOMM40* (rs2075650) identified. The y-axis shows -log_10_ P-values; x-axis shows position of genes on chromosome 19. The diamond (purple) represents the top genome-wide significant SNP. The circles represent each genotyped SNP in the region 400-kb in both directions from rs2075650; the circle color indicates pairwise linkage disequilibrium (LD) in relation to the top SNP (calculated from hg19/1000 Genomes Nov 2014 EUR). The solid (blue) line indicates the recombination rate.

Of these 29 SNPs, the strongest associations across the scans were found for rDR-L, with rs2075650 (*b* = -0.043, *p* = 5.0x10^-08^) and rs71352238 (*b* = -0.043, *p* = 7.0x10^-08^) on chromosome 19 (Fig 3C). These two SNPs passed replication criteria in the ELSA cohort, with effect sizes similar to those found in HRS (rs2075650: *b* = -0.041, *p* = 1.0x10^-04^ and rs71352238: *b* = -0.039, *p* = 2.0x10^-04^) and the. Two SNPs on chromosome 19 in the *PVRL2* (rs283815, *b*’s = -.03, *p*’s<4.0x10^-06^) and *TOMM40* (rs157582, *b*’s = -.03, *p*’s<2.0x10^-06^) genes had suggestive association with both IR-C and rDR-L. Effect sizes for these two SNPs replicated in ELSA for rDR-L (*b*’s = -.03, *p*’s<3.0x10^-03^), but not IR-C (*b*’s = -.01, *p*’s<2.0x10^-04^). Interpretation of the size of genetic effects is provided in the Supporting Information (page 4 of S3 File).

One SNP on chromosome 20, rs6125934 (*b* = -0.030, *p* = 6.0x10^-08^) approached the genome-wide cutoff for an association with rDR-L in HRS but that effect size did not replicate in ELSA (*b* = 0.004, *p* = 6.0x10^-01^). Similarly, a SNP on chromosome 20, rs6025261 (*b* = 0.079, *p* = 9.0x10^-08^) exceeded the suggestive level for an association with IR-L, but the effects did not replicate in ELSA (*b* = 0.008, *p* = 5.0x10^-01^). Several other SNPs met the criterion for suggestive association with IR-C (7 SNPs) or rDR-C (6 SNPs), but effects did not replicate in ELSA.

For the results presented in Table 3, SNPs were not pruned for linkage disequilibrium (LD, an index of the correlation between loci) because it is unknown which of the available SNPs may be the active variant for a specific gene versus serving as a marker for another variant. Fig 3 plots the surrounding SNPs and provides the pairwise LD values to the SNP (rs2075650) in the *TOMM40* region associated at the genome-wide significance level with rDR-L. As evident from the plot, the genotyping array provided enough coverage of SNPs in the region to detect three nearby SNPs in LD with rs2075650 at a level of r^2^ ≥ 0.6.

SNP heritability (SNP *h*^*2*^) in the HRS cohort was estimated for each phenotype based on the combined genetic effect across all SNPs included in the GWAS, adjusted for sex and three PCs. Estimates were significantly greater than 0 for IR-L (*h*^*2*^ = 22.4%), IR-C (*h*^*2*^ = 12.0%) and DR-L (*h*^*2*^ = 13.4%), but not for rDR-L (*h*^*2*^ = 3.4%), rDR-C (*h*^*2*^ = 6.7%) or DR-C (*h*^*2*^ = 8.6%).

### Meta-analysis results

Meta-analyses of the HRS and ELSA cohorts identified four SNPs that surpassed the genome-wide significance cut-off for rDR-L, three of which were also significantly associated with IR-C, and none of which significantly associated with rDR-C or IR-L (Table 4). All SNPs were on chromosome 19, with the *TOMM40* SNPs within 6000 base pairs of each other, and the *APOE* SNP 19,700 base pairs away. Multiple SNPs from these meta-analyses reached the suggestive threshold of 5x10^-06^ and are reported in Supporting Information (Tables A–D in S2 File). QQ and Manhattan Plots from meta-analyses for the four phenotypes are shown in Supporting Information, S5 (Figs A-D in S5 Fig) and S6 Fig (Figs A-D in S6 Fig), respectively. For characterizing the effects of the top SNPs in the *TOMM40* and *APOE* regions (reported below), we focus on SNPs that surpassed genome-wide significance and use the single top SNP to represent each of the *TOMM40* and *APOE* effects.

**Table 4:** Meta-analyses results for SNPs reaching genome-wide significance for all four phenotypes: Immediate recall level (IR-L), immediate recall change (IR-C), residualized delayed recall level (rDR-L), and residualized delayed recall change (rDR-C).

					Meta-analysis p-value			
SNP	BP	Effect allele	Gene	Direction	IR-L	IR-C	rDR-L	rDR-C
rs283815	45390333	G	*TOMM40*	++	5.1E-02	1.9E-09	8.6E-07	9.0E-03
rs71352238	45394336	G	*TOMM40*	—	4.2E-03	1.2E-06	1.0E-10	2.2E-03
rs2075650	45395619	G	*TOMM40*	—	4.7E-03	1.0E-07	5.0E-11	2.5E-03
rs157582	45396219	A	*TOMM40*	—	3.3E-02	8.3E-10	7.0E-09	5.6E-03
rs769449	45410002	A	*APOE*	—	1.9E-04	2.2E-08	3.0E-12	1.9E-02

#### Evaluation of the meta-analysis SNPs associated with rDR-L and IR-C

**Fig 4:**
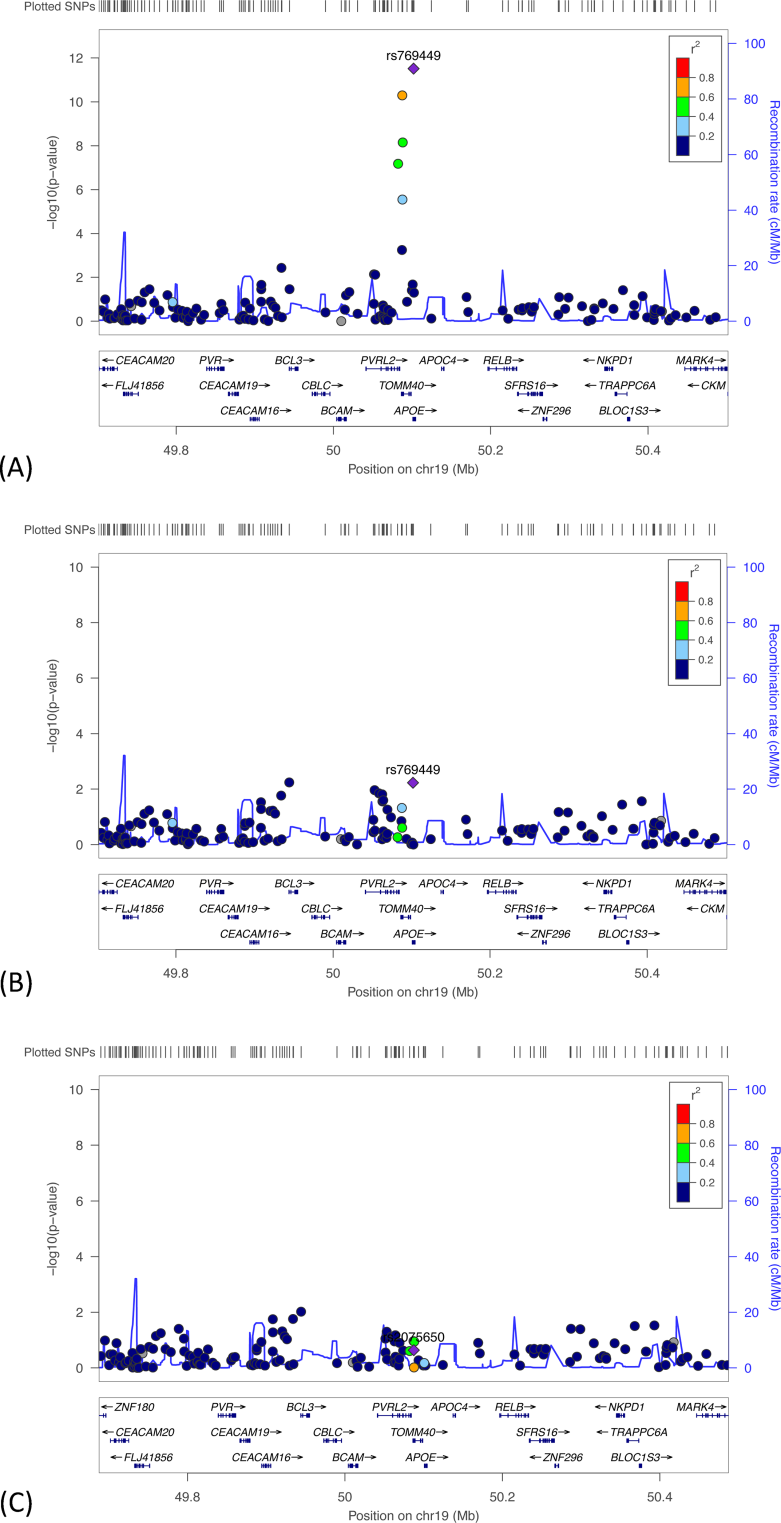
Regional plots showing meta-analyses results for rDR-L. (A) Results with the top associated SNP in the *APOE* region labeled. Two conditional meta-analyses were performed for rDR-L, (B) estimating the association with the *APOE* SNP shown (rs769449), conditioning on the top *TOMM40* SNP (rs2075650); and (C) estimating the association with the *TOMM40* SNP shown (rs2075650), conditioning on the top *APOE* SNP (rs769449). The y-axis shows -log_10_ P-values; x-axis shows position of genes on chromosome 19 with SNPs 400-kb in both directions of the SNP of interest. The diamond represents the top SNP of interest. The circles represent each genotyped SNP in this region; the circle color indicates pairwise linkage disequilibrium (LD) in relation to the top SNP (calculated from hg19/1000 Genomes Nov 2014 EUR). The solid (blue) line indicates the recombination rate.

*Evaluation of meta-analysis results for rDR-L*. In the meta-analysis of rDR-L, the top two SNPs in the *APOE* (rs769449) and *TOMM40* (rs2075650) are in LD with at r^2^ ≥ 0.6. These are represented by the top diamond and circle in Fig 4A. Because of evidence from the meta-analysis and prior findings that suggest that the effect of *TOMM40* on nonpathological cognitive decline is not independent of *APOE* effects [6], we performed a series of analyses to test this. First, we performed a meta-analysis of the GWASs that were run conditioned on the top genome-wide significant SNP in *TOMM40*, rs2075650 (Fig 4B), and found the effect of the top *APOE* SNP diminished (rs769449 p = 5.9x10^-03^). Second, we performed a meta-analysis of the GWASs that were run conditioned on the top genome-wide significant SNP in *APOE*, rs769449 (Fig 4C), and found the effect of the top *TOMM40* SNP diminished (rs2075650 p = 0.23) to a non-significant effect.

Third, we analyzed rDR-L, conditioned on *APOE* e4. SNP rs769449 resides in *APOE*, and is in high pairwise LD with the SNPs that determines the *APOE* e4 isoform, rs429358 (r2 = 0.779), as calculated using the Phase 3 of the 1000 Genomes Project and CEU reference panel [73]. The presence of *APOE* e4, and thus *APOE* genotype, was not determined from directly genotyped SNPs in HRS or ELSA, and thus it was not included in the primary analyses of this study. However, because the two non-synonymous SNPs that characterize the isoforms (rs429358 and rs7412) were inferred through imputation with estimated certainties ≥.997 in HRS [74], and have demonstrated high agreement with directly genotyped SNPs in other studies [75, 76], we were able to conduct additional evaluation of our finding using the isoforms in the HRS cohort. Details of the imputation process are provided by HRS [77, 78] and summarized in the Supporting Information (see page 1, in S3 File). When performing linear regression for the effects of *TOMM40* rs2075650 on rDR-L, with *APOE* e4 in the model, and covariates (sex, 3 PCs), we found that the effect of *TOMM40* remained (b = -0.0259, SE = 0. 0108, p = .017) with effect of e4 also significant (b = -0.0295, SE = 0.0124, p = .017).

Fourth, we tested the effects of *TOMM40* on rDR-L in other *APOE* genotypes to examine effects beyond e4. Results of each model predicting rDR-L are provided in the Supporting Information (Table A in S3 File). Most notably, there was an effect of the rs2075650 G allele on rDR-L within e3/e3 individuals (b = -0.0390, SE = 0.0180, p = .031).

**Fig 5:**
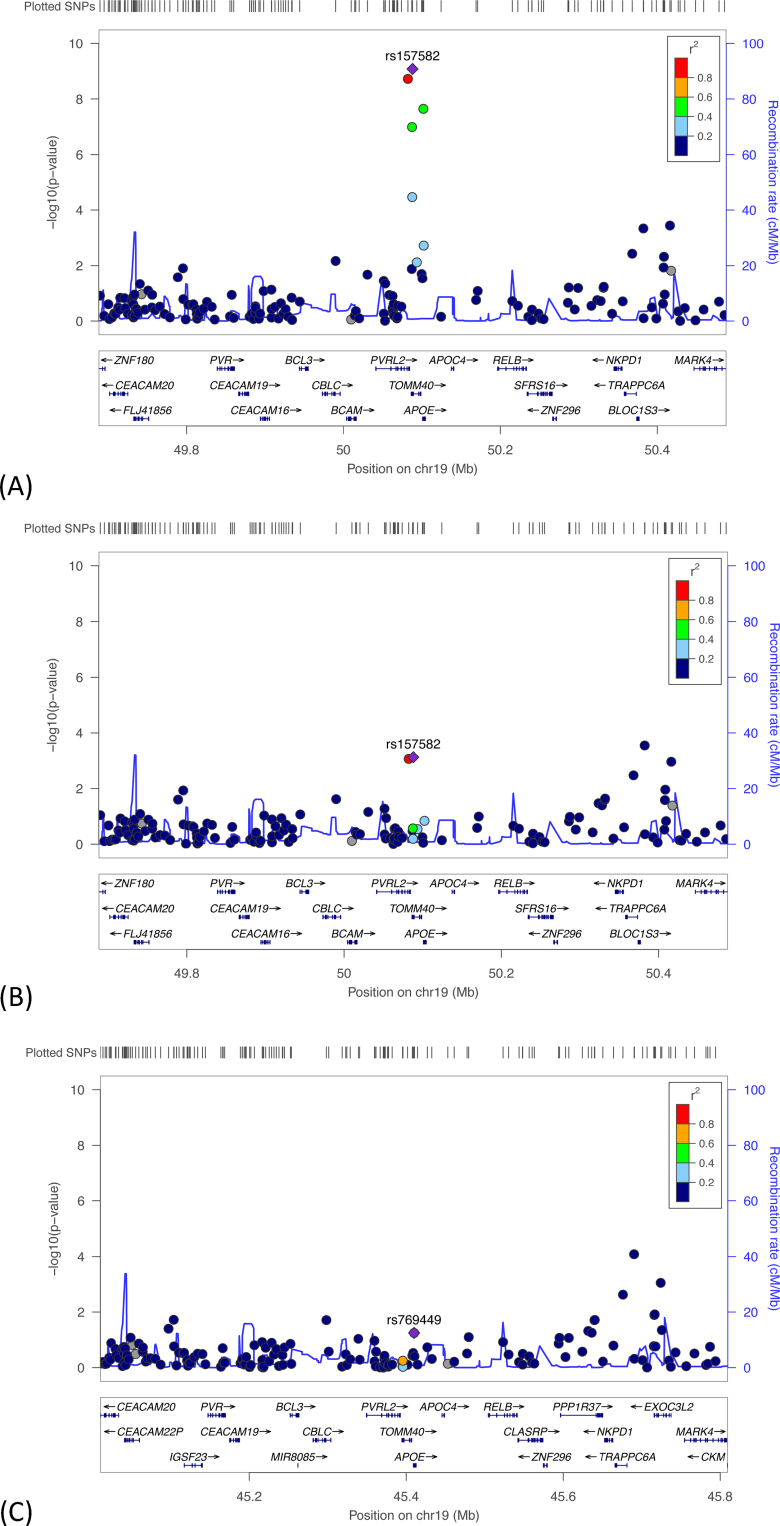
Regional plots showing results of the meta-analyses of IR-C. (A) Meta-analysis results with the top SNP in *TOMM40* (rs157582) shown; (B) results when estimating the association with the *TOMM40* SNP shown (rs157582), conditioning on the top *APOE* SNP (rs769449); and (C) results when estimating the association with the *APOE* SNP (rs769449), conditioning on the top *TOMM40* SNP (rs157582). The y-axis shows -log_10_ P-values; x-axis shows position of genes on chromosome 19 with SNPs 400-kb in both directions of the SNP of interest. The diamond represents the top SNP of interest. The circles represent each genotyped SNP in this region; the circle color indicates pairwise linkage disequilibrium (LD) in relation to the top SNP (calculated from hg19/1000 Genomes Nov 2014 EUR). The solid (blue) line indicates the recombination rate.

*Evaluation of meta-analysis results for IR-C*. From the meta-analysis of IR-C, the top two identified SNPs were in *TOMM40* (rs157582) and *APOE* (rs769449), and in LD at r^2^ ≥ 0.8. Fig 5A shows the results of this meta-analysis, with the top associated SNP labeled. We examined whether the effects of the top two SNPs on this phenotype were independent by running a series of analyses. First, we performed a meta-analysis of the GWASs, conditioned on the top genome-wide significant *APOE* SNP (rs769449), to examine the change in effect of *TOMM40* SNP rs157582 (Fig 5B). Compared to the joint analysis (Fig 5A), the conditional analysis found the association between IR-C and rs157582 diminished (p = 7.4x10^-04^), as did the effect of another *TOMM40* SNP (rs71352238 p = 8.5x10^-04^). Second, we performed a meta-analysis of the GWASs, conditioned on the top genome-wide significant *TOMM40* SNP (rs157582), to examine the change in effect of *APOE* SNP rs769449 (Fig 5C). The association between IR-C and *APOE* was no longer significant (rs769449, p = 0.06).

Third, we constructed linear regression models using the HRS cohort, predicting IR-C from the e4 allele, adjusted for covariates, and found a significant but small effect of e4 (b = -0.032, SE = 0.009, p = .001). Next, in a model predicting IR-C from both the *TOMM40* SNP, rs157582 (b = -0.031, SE = 0.013, p < .0001) and *APOE* e4 (b = -0.008, SE = 0.012, p = .53), the effect of *TOMM40* remained while the effect of *APOE* was reduced and no longer significant.

Lastly for IR-C, we tested the effects of the *TOMM40* rs157582 A (risk) allele within each of the *APOE* genotypes. The models showed that there were significant effects of the rs157582 A allele on IR-C within the e3/e3 (b = -0.0310, SE = 0.016, p = .047) and e2/e3 strata (b = -0.0477, SE = 0.022, p = .032). Results of each model predicting IR-C are provided in the Supporting Information (Table B in S3 File).

### SNP effects on rDR-L and IR-C across age decades

**Fig 6:**
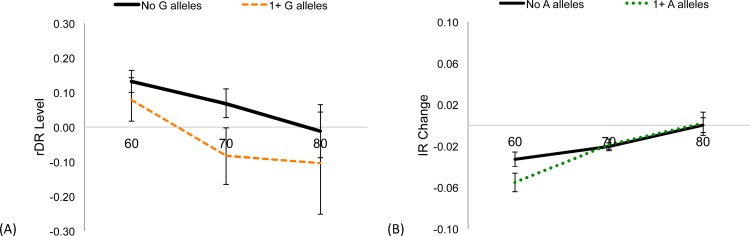
Plot of effect sizes of top *TOMM40* risk alleles against age, adjusted for *APOE* e4 presence. (A) Effect of the G allele in rs2075650 on rDR-L and (B) Effect of the A allele in rs157582 on IR-C. The lines represent age-related trajectories of the phenotype for individuals who have no risk alleles (solid line) or 1 or more risk allele (dashed line).

Because our phenotypes reflected age-related trajectories, and due to evidence that genes have stronger effects on cognitive performance in older ages [27, 29], we evaluated whether the effect of *TOMM40* on rDR-L and IR-C independent of *APOE* e4 differs at older age decades. Using the HRS cohort and mixed effects modeling, additional splines were fit with the knot point at age 70 and age 80, adjusted for *APOE* e4 carrier status, sex, and 3 PCs. When plotting the age-related rDR trajectories were stratified by presence of the rs2075650 ‘G’ allele, we find that in older ages, there were larger differences in the effects of the *TOMM40* allele that were independent of APOE e4 (Fig 6A). In contrast, for IR change, the effect of the *TOMM40* rs157582 ‘A’ allele that was independent of *APOE* e4 was evident only at age 60, when the A allele associated with a larger, negative change score (i.e., steeper decline); in older age decades, there were no differences in effects of the A allele on IR change (Fig 6B).

### Gene network analysis

We evaluated the networks and molecular pathways including the relationships between *TOMM40*, *APOE*, and memory phenotypes from the IPA library of canonical pathways, disease processes, and biological processes, as well as customized pathways that were within the network. IPA analyses was seeded with established risk genes for AD (listed via AlzGene: *APOE*, *BIN1*, *CLU*, *ABCA7*, *CR1*, *PICALM*, *MS4A6A*, *CD33*, *MS4A4E*, *CD2AP*) and from the current study, as associated with rDR-L and IR-C, in order to elucidate relationships between genes and the variants involved in AD-related processes. The network (S7 Fig), shows that *TOMM40* has possible interactions or functions in conjunction with several genes (i.e., *APOE*, *BAX*, *INSR*, *APP*, *VDAC4*, *FOXB1*, *SNCA*, *HSD17B10*, *HSPA4*) in the pathway of influence on verbal memory, episodic memory, AD, cognitive impairment, memory deficits, and reduced hippocampal volume. Some of these pathways likely involve other genes (e.g., *CLU*, *PICALM*, *BDNF*, *CD33*, *CEBPB*, *TNFRSF19*, *ABCA7*, *BIN1*, *PPARGC1A*) with upstream influence from *F2*. The network also shows interactions between *APOE* and other genes in nearly every pathway to a memory and cognition-related outcome.

## Discussion

In this study, we used a population-based sample repeatedly assessed on verbal memory to characterize individual trajectories of change over age, giving greater precision in assessing level and decline of memory ability than available in most other studies. Trajectories were constructed to reflect immediate and delayed recall after age 60, an age at which other longitudinal studies suggest consistent decline in memory ability occurs. One SNP (rs2075650) on chromosome 19, in the *TOMM40* region, associated at the genome-wide significant level with the phenotype of delayed recall level that is independent of immediate recall. This finding replicated in a completely independent sample and remained in our subsequent meta-analysis. The meta-analysis on delayed recall level also identified two other *TOMM40* SNPs (rs71352238, rs157582) and an *APOE* SNP (rs769449) with genome-wide significant associations. Additional evaluation of the *TOMM40* and *APOE* SNPs indicated that the GWAS signal for delayed recall level was mostly attributed to *APOE*; however, there were effects of the *TOMM40* variant independent of e4. The meta-analysis on the separate phenotype of immediate recall change identified genome-wide significant SNPs in both genes (*TOMM40* rs157582 and rs71352238; *APOE* rs769449). Additional evaluation indicated that *TOMM40* had not only stronger effects than APOE on immediate recall change, but also effects independent of e4. Several other SNPs that reached the suggestive association level in the present study reside within genes associated with memory phenotypes in other studies. More critically, our results underscore the importance for specificity in phenotypes when assessing the genetic influence on components of memory abilities.

### Discrete components of verbal memory

An innovation of this study was to separate the components involved in different aspects of recall. Whereas immediate recall reflects initial encoding and rehearsal, our residualized-delayed recall score, created by partialling the immediate score from the delayed score, is a distinct phenotype reflecting the “pure” retrieval component of memory (depicted in Fig 1). A similar approach has been used previously to create a pure measure of delayed memory function [79]. In the current study, separating the components provided greater insight into the basis for changes in verbal memory over age. About half of the variation in decline in DR after age 60 can be attributed to decline in IR (See Supporting Information, Tables A-B in S1 File). Also, after adjusting for what is initially encoded, there are small individual differences in what people can retrieve and even smaller individual differences in how people decline in retrieval.

### Genetic specificity for aging-related changes in verbal memory components

The overarching goal of this study was to examine the complexity of verbal memory abilities over age, and whether genetic variants associate uniquely with specific components of verbal memory, represented by four phenotypes. Our finding with regard to rDR level supports work from prior cognitive aging studies. Taken together, although rs2075650, in the *TOMM40* region, associates with rDR level in our study, and with episodic memory (p<5.0x10^-08^) and late-onset Alzheimer disease (LOAD) in other studies, that effect on verbal information retrieval detected through GWAS is primarily driven by linked *APOE* variants [6]. With regard to IR change, our findings indicate that *TOMM40* plays a larger role, specifically, in decline of verbal learning after age 60. Further, our analysis showed that there are unique effects of *TOMM40* beyond *APOE* e4 effects on both level of delayed recall prior to age 60 and decline in immediate recall after 60.

With the IR-C finding, additional evaluation of the effect of the *TOMM40* SNP (rs157582) indicates that it has significant effects outside of e4. Upon cursory assessment with *TOMM40* being 16 kb upstream of e4, conclusions could be drawn that the *TOMM40* SNP simply serves as a marker for the e4 effect; however, several critical pieces of information suggest otherwise. First, the *APOE* SNP rs769449 resides 2 kb from e4 and is in high LD with it (r2 = .799) such that if the effect of *TOMM40* on IR-C were attributable solely to *APOE*, we would have observed the effect of *TOMM40* disappear when it was included in a model with rs769449 predicting IR-C. Yet, the effect of *TOMM40* remained statistically significant whereas the effect of rs769449 did not. Second, if the signal were attributable solely to *APOE*, when the effect of the *TOMM40* SNP was conditioned on the e4 allele (inferred from rs429358), the effect size of *TOMM40* would have diminished. Conversely, the effect size of *TOMM40* remained the same while the effect of e4 diminished to non-significance. Several additional observations point towards an independent *TOMM40* effect. One relates to linkage disequilibrium (LD) and that the *TOMM40* SNP (rs157582) is in imperfect LD with the *APOE* SNPs (r2 = .456 with rs769449; r2 = .590 with rs429358 [73]). Therefore, these SNPs cannot serve as interchangeable proxies for one another. Additional support comes from the observed associations with late onset Alzheimer disease (LOAD). When both rs157581 (residing 500 bp from rs157582 on *TOMM40*) and rs429358 were directly genotyped and tested in separate additive logit models for associations with LOAD, they showed different degrees of effect (ORs of 2.9 and 4.1, respectively [80]). Third, and perhaps most surprising, is the finding that rs157582 showed statistically significant effects among e3/e3 individuals, but not those who carry e4. Taken together, these findings suggest there is some degree of effect of *TOMM40* that occurs independently of *APOE* e4.

Our findings are in line with several recent studies that have implemented newer methods to evaluate *APOE*-*TOMM40* haplotype associations [81] and found *TOMM40* variants influence memory performance and dementia risk beyond that conferred by the *APOE* e4 variant [82–94]. For example, individuals with an *APOE* e3-*TOMM40* VL haplotype developed LOAD an average of seven years earlier than those with the *APOE* e3-*TOMM40* S haplotype (70.5±1.2 years vs. 77.6±2.1 years, p = 0.02 [95]). This indicates that the *TOMM40* polymorphism significantly influences age of disease onset for those with an e3 allele, which had been considered a low risk allele prior to these haplotype studies. Other studies have found that within *APOE* e3/e3 individuals, the VL variant of the *TOMM40* polymorphism is associated with lower performance on episodic memory [84, 94]. Our study showed there were differences in effects of *TOMM40* on delayed verbal memory and immediate recall change depending on *APOE* genotype, with notable effects among e3/e3 individuals. Given these findings, devising a study in which the *TOMM40* poly-T length polymorphisms (also known as the ‘523’ repeat) have been characterized to test the association between *APOE* e3/e3-*TOMM40* variant haplotypes with delayed verbal recall level and immediate recall change could provide further understanding of these findings.

Second, and more relevant to genetic specificity for verbal memory, prior GWAS studies use phenotypes (such as a TICS total score) that aggregate multiple cognitive domains, including crystallized ability, fluid ability, speed, and episodic memory. Since these processes differ in their phenotypic trajectories over age [96, 97] and genetic structure [98, 99], such aggregation may obscure genetic associations specific to memory. Our findings suggest even greater specificity, that variation in the *TOMM40/APOE* region is associated with level of ability in the retrieval component of verbal memory and change in initial encoding and rehearsal. Given the key importance of delayed recall in discriminating degrees of cognitive impairment, partialling out the phenotype to reflect true retrieval provides a more refined measure with which to study and potentially link underlying genetic associations with impairment. The interpretation of our findings would be clarified further by following up these results in a sample that has genotyping data and assessment of memory in non-verbal domains and probing of encoding by recognition as well as recall.

### Biological implications and specificity

Our findings that *TOMM40* and *APOE* associate with level of delayed recall and change in immediate recall have plausible biological mechanisms. An examination of gene interactions and protein expression through gene network analyses identified possible pathways from both genes to AD and memory-related conditions. For example, cognitively intact, older *APOE* e4 carriers have been shown to exhibit greater age-related reductions in hippocampal volume [100] and reduced functional activity in the right hippocampus [101]. In another study of healthy older adults (ages 60 to 87), lower hippocampal volume was associated with lower recall abilities among *APOE* e4 carriers in combination with the *TOMM40* rs11556505 ‘T’ (r = 0.28, p<0.01, R^2^ = 0.08) and rs2075650 (the SNP in our study) ‘G’ (r = 0.28, p<0.01, R^2^ = 0.08) “risk” alleles [89]. This suggests that the additional effects of *TOMM40* “risk” alleles on recall abilities, beyond effects of *APOE*, may be mediated via brain morphology.

Although verbal memory components may share some genetic underpinnings and biologic pathways, prior research and our findings support the notion that phenotypic specificity remains critically important. In a prior study, left hippocampal body volume was shown to be associated with delayed verbal memory (r = -0.17, p < .05), but not immediate memory among healthy older adults, ages 55 to 83 [16]. In another study, in which a residualized DR score was also used to remove the influences of IR, hippocampal size was associated with rDR performance, but not with IR [79]. Numerous findings have correlated verbal memory with other regions of the medial temporal lobes, outside of the hippocampus, although more recent evidence from functional neuroimaging studies of AD patients indicates that this is driven by learning rather than retrieval processes [15]. These findings suggest the need for further investigation of the relationship between hippocampal size, other regions of the medial temporal lobe, specific component of verbal memory, and the associated genetic profile to provide greater insight into biological mechanisms for age-related differences in memory abilities.

### Repeated measures and other study strengths

This study addressed several limitations of prior research aimed at identifying genetic loci associated with memory decline, including the use of cross-sectional data to estimate age-related change and using an index of delayed memory that is confounded with immediate. We harnessed repeated measures from a large population-based study and created distinct phenotypes that better reflect the complexity of verbal memory ability and change over time. Results from longitudinal studies of episodic memory can be limited by practice effects [26], but this is of less concern in HRS because assessments were given every two years using alternate forms. We reduced the potential impact of selective attrition [102] by including individuals who had participated in HRS at least two occasions within 16 assessment years (1996 to 2012).

### Limitations

This study had several limitations. First, our measure of verbal memory was based on a single presentation of a word list administered by a lay-interviewer and likely contains greater measurement error than other approaches using repeated presentations of a word list or multiple memory measures. However, this would be expected to introduce noise rather than false positive results. This illustrates a significant challenge for GWAS study designs: achieving samples sufficiently large to detect the expected effect sizes often means a trade-off in measurement intensity. By using a repeated measures design in a large sample we were able to characterize individual trajectories of age-related change and increased power compared to cross-sectional designs [103], but we still had limited power to detect additional SNP associations unique to the components of verbal memory. Third, variance in rDR change over time was particularly small. This partially explains the difficulty in detecting associations between genetic loci and change. Fourth, there are a variety of covariates that could have been included that may mediate the association between genetic effects and later life memory–including indices of health and socioeconomic status. We intentionally did not incorporate these in the analyses because it is arguable that these are outcomes rather than causes of memory ability and decline (for more discussion, see page 5 of S3 File). Further, for the GWAS in particular, there may be genetic pleiotropy between memory ability and these variables, especially educational attainment. Fifth, in examining the joint effect of *TOMM40* and *APOE* on rDR-L and IR-C, we were not able to directly account for the effects of *APOE* e4, nor were we able to examine haplotype effects. Thus, our analyses cannot confirm causality between *TOMM40* and phenotypes, nor can we confirm the independence of the effect of *TOMM40* given the proximity to other susceptibility genes. For further confirmation, the finding warrants replication with a sample in which the genotyped *APOE* alleles are available, as well as the *TOMM40* poly-T variant.

## Conclusion

The current study provides evidence of genetic underpinnings for specific components of aging-related verbal memory performance, delayed recall at age 60 that is unconfounded with attention or memory span, and decline in immediate recall after age 60. The results underscore the importance of studying phenotypes that represent distinct components of memory, or other aspects of cognition, when assessing genetic influences. One identified genetic variant, a SNP in the *TOMM40* region, has biological plausibility for its effect on delayed recall beyond its association with *APOE* e4 risk because of findings linking *TOMM40*, hippocampal formation and volume, and delayed recall. With regard to change in immediate recall ability, there is evidence for the genetic effects of both *TOMM40* and *APOE*, as well as independent effects of *TOMM40* among individuals who do not carry the *APOE* e4 allele. These findings need confirmation in other samples, but support the existence of differential effects of *TOMM40* and *APOE* on specific components of verbal memory.

## Supporting information

S1 Fig (PDF)

S2 Fig (PDF)

S3 Fig (PDF)

S4 Fig (PDF)

S5 Fig (PDF)

S6 Fig (PDF)

S7 Fig (PDF)

S1 File Supporting information. (PDF)

S2 File Meta analyses. (PDF)

S3 File Additional evaluation. (PDF)
