# Supplementary material for: Genetic variants specific to aging-related verbal memory: Insights from GWASs in a population-based cohort
Source: PLoS One. 2017 Aug 11;12(8):e0182448. doi: 10.1371/journal.pone.0182448 (PMC5553750; doi:10.1371/journal.pone.0182448)
Supplement: S1 Fig — (PDF) [file pone.0182448.s001.pdf]

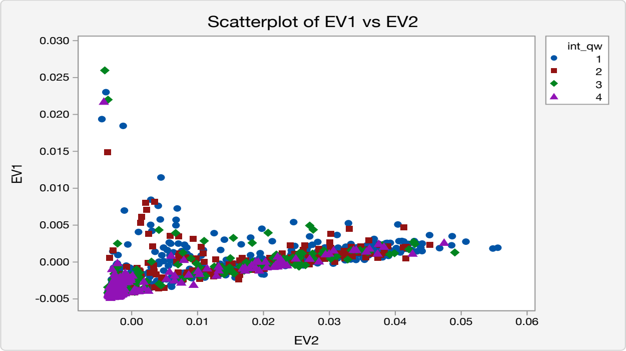

A.

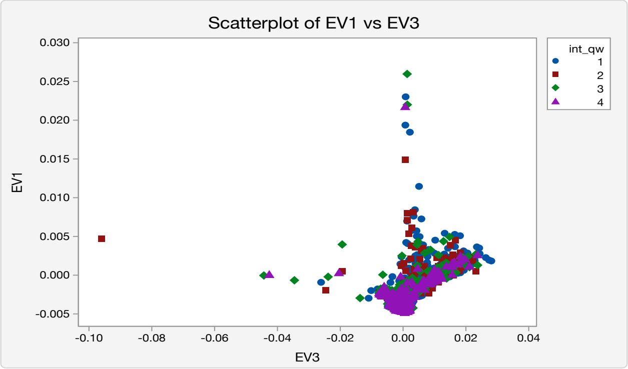

B.

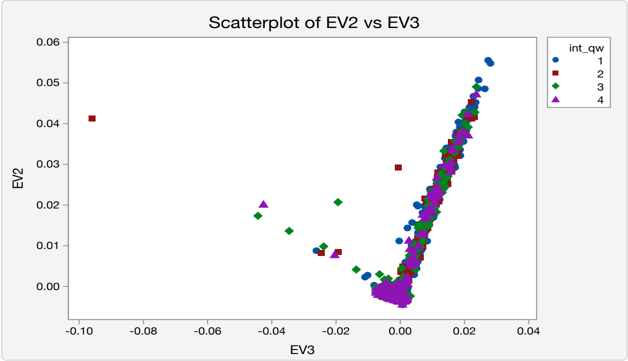

C.

Fig S1. Eigenvalues (EV) for principal components 1 through 3 are shown, comparing the distribution by quartiles of immediate recall level (1=highest quartile score to 4=lowest quartile score), comparing (A) EV1 and EV2, (B) EV1 and EV3, and (C) EV2 and EV3. The plots indicate no overarching pattern by which sample admixture influences verbal memory performance. A single outlier (red square in the far right of Figs B and C) was removed during data quality control processes.
