# Supplementary material for: Genetic variants specific to aging-related verbal memory: Insights from GWASs in a population-based cohort
Source: PLoS One. 2017 Aug 11;12(8):e0182448. doi: 10.1371/journal.pone.0182448 (PMC5553750; doi:10.1371/journal.pone.0182448)
Supplement: S2 Fig — (PDF) [file pone.0182448.s002.pdf]

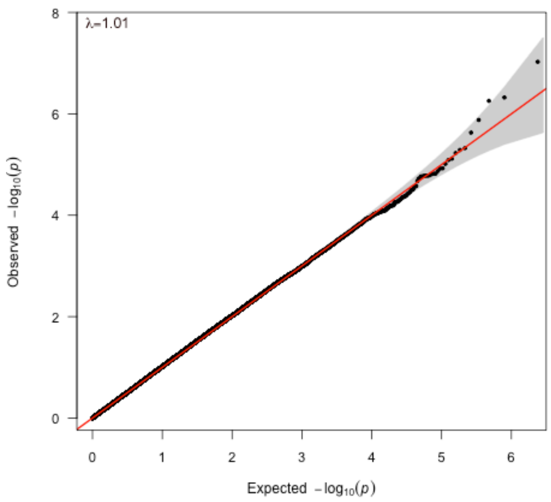

A. IR-L

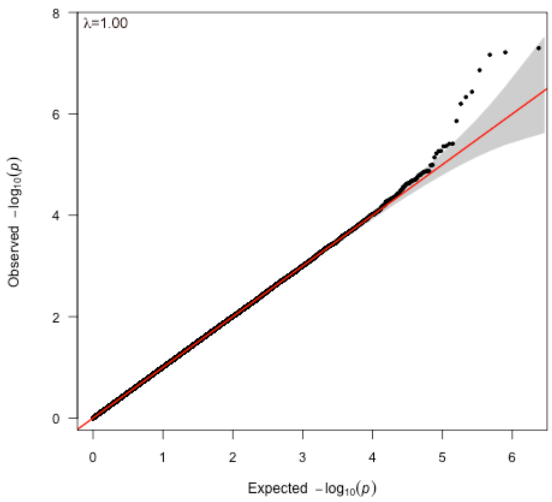

C. rDR-L

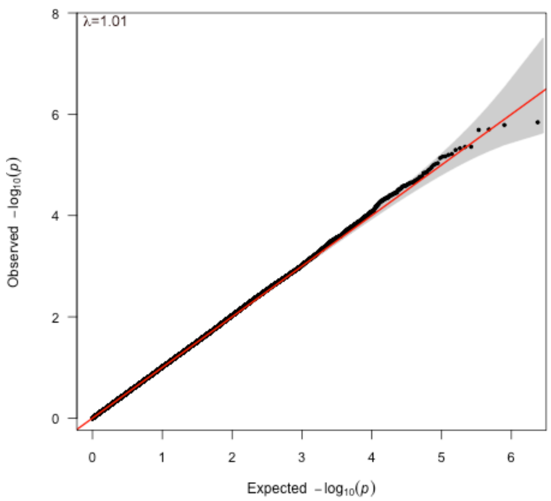

B. IR-C

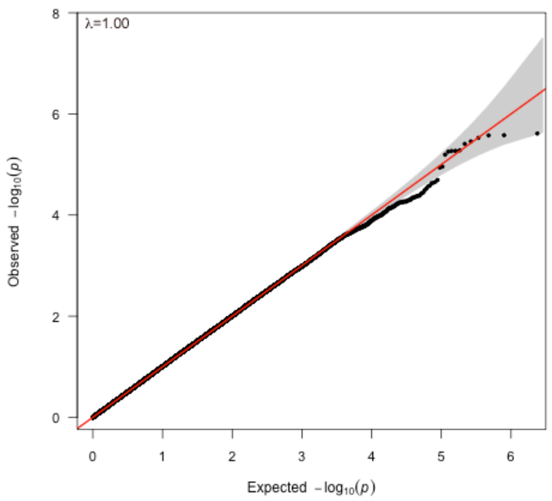

D. rDR-C

Fig S2. QQ plots for each phenotype from GWAS on the HRS genetic sample.
(A) Level of Immediate Recall (IR-L); (B) Change in Immediate Recall (IR-C); (C) Level of Residualized Delayed Recall (rDR-L); (D) Change in Residualized Delayed Recall (rDR-C). QQ plots show the observed plotted against the expected p-values for each GWAS run. The dots represent the observed data and the straight line represents the expectation under the null hypothesis of no association. The gray shaded area shows the 95% confidence interval. When the majority of observed values (dots) do not deviate from the 95% confidence area along the trajectory of the expected line, inflation in p-values (type 1 error) is not suspected. The solid (red) line is the expectation under the null hypothesis of no association. Lambda value (λ) for each scan was calculated by dividing the median of the observed chi-square statistic by the expected median in the absence of stratification.
