# Supplementary material for: Genetic variants specific to aging-related verbal memory: Insights from GWASs in a population-based cohort
Source: PLoS One. 2017 Aug 11;12(8):e0182448. doi: 10.1371/journal.pone.0182448 (PMC5553750; doi:10.1371/journal.pone.0182448)
Supplement: S3 Fig — (PDF) [file pone.0182448.s003.pdf]

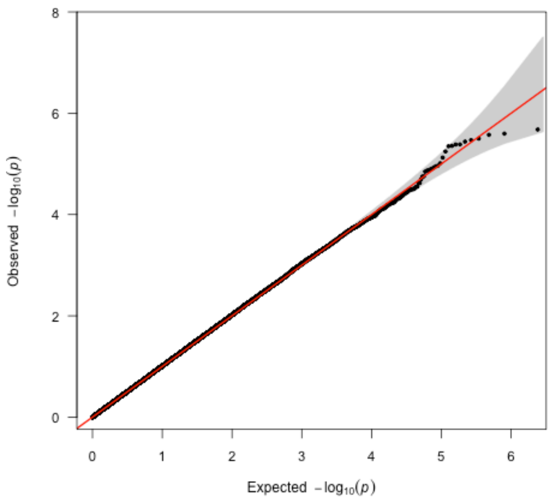

A. IR-L

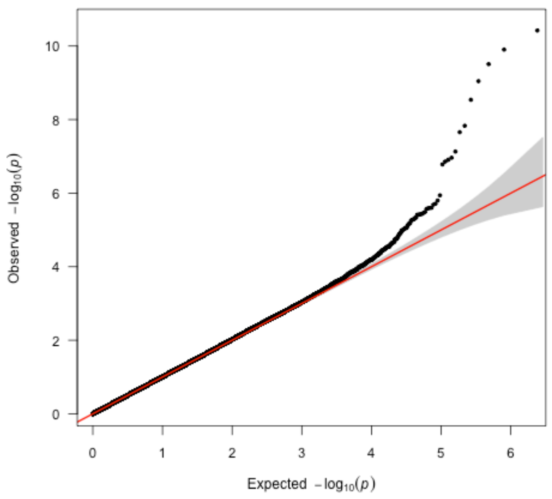

C. rDR-L

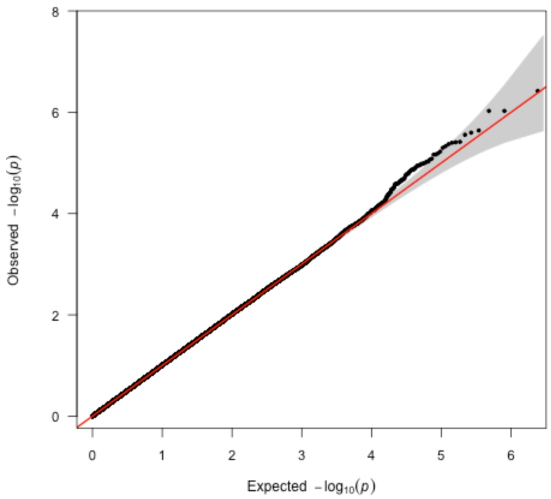

B. IR-C

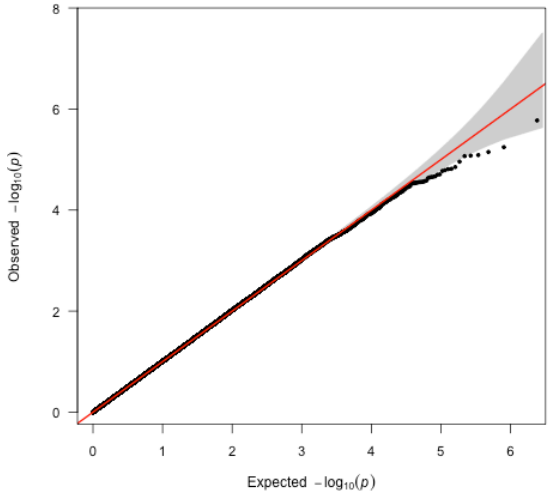

D. rDR-C

Fig S3. QQ plots for the ELSA replication sample for all phenotypes: (A) immediate recall level (IR-L) and (B) immediate recall change (IR-C) and (C) residual delayed recall level (rDR-L) and (D) residual delayed recall change (rDR-C). QQ plots show the observed plotted against the expected p-values for each GWAS run. The dots represent the observed data and the straight line represents the expectation under the null hypothesis of no association. The gray shaded area shows the 95% confidence interval. When the majority of observed values (dots) do not deviate from the 95% confidence area along the trajectory of the expected line, inflation in p-values (type 1 error) is not suspected.
