# Supplementary material for: Genetic variants specific to aging-related verbal memory: Insights from GWASs in a population-based cohort
Source: PLoS One. 2017 Aug 11;12(8):e0182448. doi: 10.1371/journal.pone.0182448 (PMC5553750; doi:10.1371/journal.pone.0182448)
Supplement: S6 Fig — (PDF) [file pone.0182448.s006.pdf]

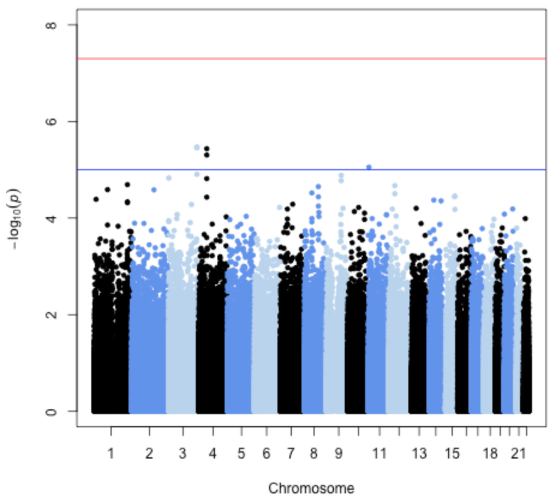

A. IR-L

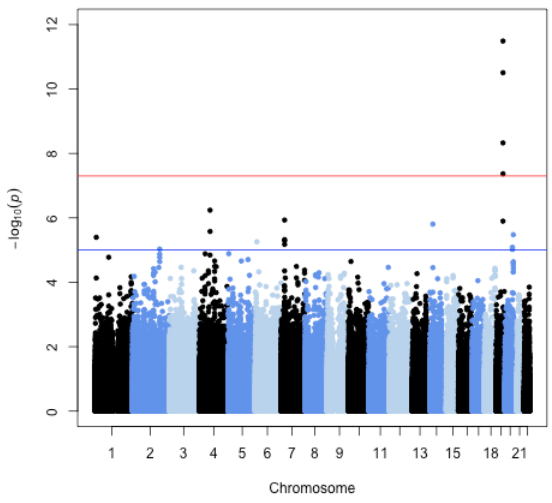

C. rDR-L

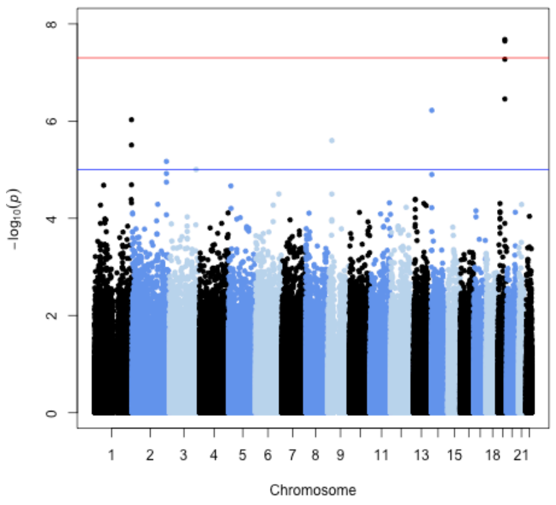

B. IR-C

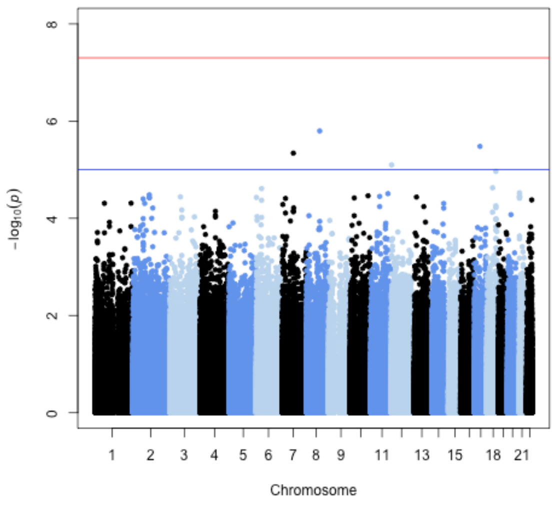

D. rDR-C

Fig S6. Plots show p-values for all SNP associations for the meta-analysis, for all phenotypes: (A) immediate recall level (IR-L) and (B) immediate recall change (IR-C) and (C) residual delayed recall level (rDR-L) and (D) residual delayed recall change (rDR-C). The upper (red) horizontal line demarcates the threshold of p= -log($5.0x10^{-08}$) and the lower (blue) horizontal line demarcates p=-log($1x10^{-05}$) for SNPs based on their chromosomal position (x-axis).
