# Supplementary material for: Genetic variants specific to aging-related verbal memory: Insights from GWASs in a population-based cohort
Source: PLoS One. 2017 Aug 11;12(8):e0182448. doi: 10.1371/journal.pone.0182448 (PMC5553750; doi:10.1371/journal.pone.0182448)
Supplement: S7 Fig — (PDF) [file pone.0182448.s007.pdf]

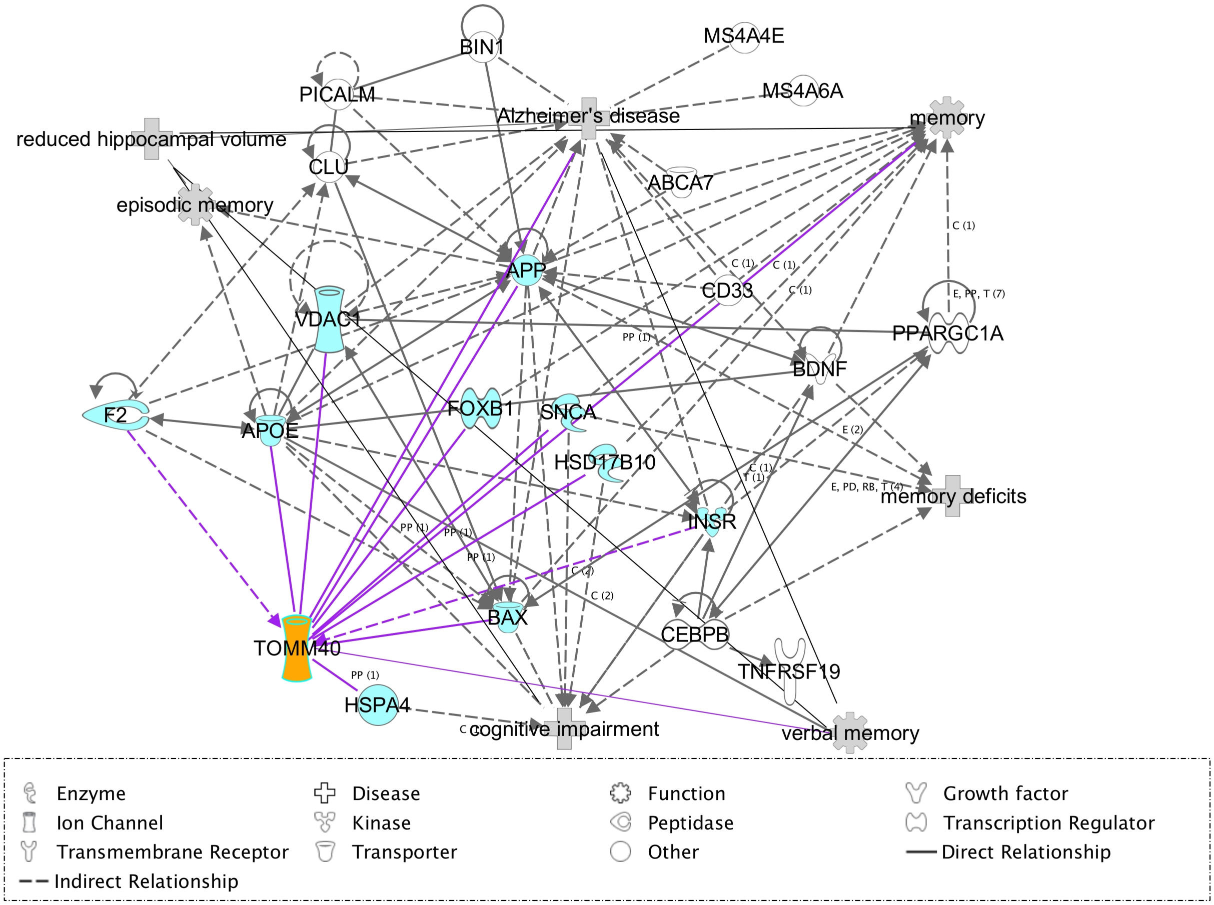

Fig S7. Network derived through pathway analysis containing *TOMM40* (orange), previously-known AD genes including *APOE*, and others identified as involved in canonical pathways. Genes most proximal to *TOMM40* are linked with darker (purple) connectors. The relationship between two nodes is denoted as direct (solid line) or indirect (discontinuous line). Arrows indicate modulatory roles of proteins and/or endogenous chemicals on the expression of other proteins. Shapes of the nodes indicate distinct functions shown in the key. The curved line and arrow indicate intracellular and extracellular translocation, respectively. Shaded (gray) nodes depict diseases or conditions, as labeled.
