# Supplementary material for: Genetic variants specific to aging-related verbal memory: Insights from GWASs in a population-based cohort
Source: PLoS One. 2017 Aug 11;12(8):e0182448. doi: 10.1371/journal.pone.0182448 (PMC5553750; doi:10.1371/journal.pone.0182448)
Supplement: S1 File — (PDF) [file pone.0182448.s008.pdf]

Additional Analyses on Overlap of the Correlated Phenotype: DR-C

To further examine the overlap between IR-C and DR-C, and DR-C and rDR-C, we conducted additional analyses. Change in DR scores were regressed on change in IR and rDR scores, adjusted for baseline age (age of first assessment after 60), years of follow-up in the study (M=8.47, SD=5.10), and sex. Change in IR and rDR scores accounted for 49% and 41% of the variation in change in DR scores after age 60, respectively. More detail is provided in the tables below.

Table A. Variation in DR decline accounted for by IR and rDR decline: Regressing DR on IR change scores, adjusted for age, years of follow-up, and sex

	**B**	**SE**	**P**
Age (baseline)	-0.003	0.002	0.11
Years of follow-up	-0.014	0.002	<.0001
Sex (1=M)	-0.111	0.024	<.0001
Change in IR	0.832	0.006	<.0001
**F-value**			**5026.40**
**Adjusted R^2**			**0.49**

Table B. Variation in DR decline accounted for by IR and rDR decline: Regressing DR on rDR change scores, adjusted for age, years of follow-up, and sex

	**B**	**SE**	**P**
Age (baseline)	-0.017	0.002	<.0001
Years of follow-up	-0.053	0.003	<.0001
Sex (1=M)	-0.276	0.026	<.0001
Change in rDR	0.873	0.007	<.0001
**F-value**			**3656.92**
**Adjusted R^2**			**0.42**

Ad Hoc Analyses: GWASs on delayed recall level (DR-L) and delayed recall change (DR-C)

Because immediate recall (IR) and delayed recall (DR) are phenotypically correlated, we created an independent phenotype of residualized-delayed recall (rDR). Thus, IR represents primarily the processes of acquisition and rehearsal whereas rDR represents primarily the processes of long term memory and retrieval. To provide context for interpreting the results for our four phenotypes (IR level (IR-L), IR change (IR-C), rDR level (rDR-L), and, rDR change (rDR-C)), we ran GWASs on DR level (DR-L) and DR-change (DR-C).

Results

Table C provides the effect sizes and p-values for all six phenotypes for the top GWAS hits associated with IR-L, IR-C, rDR-L, and rDR-C. For the one SNP (chr 19, rs2075650) with genome-wide significance for its association with rDR-L (b=-0.043, p=5x10^{-08}) the finding was specific to rDR-L and did replicate for the uncorrelated phenotype of IR-L (b=-0.032, p=1x10^{-01}) but did for the correlated phenotype of DR-L (b=-0.104, p=6x10^{-05}). Across many of the other listed SNPs, the effect sizes for the DR phenotypes are larger than for rDR, but there are also larger p-values.

Discussion

DR-L and DR-C are complex phenotypes, requiring intact initial encoding and rehearsal as well as later retrieval. Disruption in any of these processes will interfere with DR performance. More phenotypic variation is seen for DR scores (Table 2 in article), but the genetic etiology may be more complex, reflected in the greater heterogeneity and larger p-values observed for DR than for rDR. Therefore, results of these ad hoc analyses support our approach of using rDR to isolate the aspects of delayed retrieval in DR that do not depend on IR.

Table C. Strongest associated SNPs from GWASs in the HRS sample, for immediate recall level (IR-L) and change (IR-C) and residual delayed recall level (rDR-L) and change (rDR-C), with comparison values for the correlated phenotypes of delayed recall level (DR-L) and delayed recall change (DR-C)

				IR-L		DR-L		rDR-L		IR-C		DR-C		rDR-C	
Chr.	SNP	Location	Allele	Beta	P-value	Beta	P-value	Beta	P-value	Beta	P-value	Beta	P-value	Beta	P-value
1	rs11804244	163212633	G							-0.029	4.E-06	-0.021	3.E-03	0.001	3.E-01
1	rs6704030	163218278	G							-0.029	5.E-06	-0.021	3.E-03	0.001	3.E-01
2	rs12463410	207715608	A							-0.054	2.E-06	-0.044	5.E-04	0.001	5.E-01
6	rs9504162	4599442	A	-0.013	4.E-01	-0.063	1.E-03	-0.028	4.E-06						
6	rs7766458	54140128	C	-0.095	5.E-06	-0.111	1.E-05	-0.017	3.E-02						
6	rs12195716	79535412	G							0.005	3.E-01	-0.012	5.E-02	-0.005	4.E-06
6	rs2174743	79591805	A							0.005	4.E-01	-0.012	5.E-02	-0.005	4.E-06
6	rs7756858	79619968	G							0.005	4.E-01	-0.012	5.E-02	-0.005	2.E-06
6	rs1572585	79690576	A							0.005	4.E-01	-0.012	4.E-02	-0.005	3.E-06
6	rs9372456	116592956	G							0.028	1.E-06	0.013	4.E-02	-0.001	3.E-01
6	rs10581090	116658967	A							0.026	4.E-06	0.011	7.E-02	-0.001	3.E-01
6	rs147391086	157984507	A	-0.153	1.E-06	-0.134	5.E-04	-0.010	9.E-01						
6	rs150510877	157994175	A	-0.159	6.E-07	-0.139	3.E-04	-0.001	9.E-01						
12	rs10880835	46079245	C							-0.001	9.E-01	0.012	6.E-02	0.005	3.E-06
12	rs35162469	46081065	A							-0.001	9.E-01	0.011	7.E-02	0.005	3.E-06
13	rs9510784	24188270	A	-0.027	7.E-02	-0.072	7.E-05	-0.025	4.E-06						
15	rs4076414	86441452	C	0.009	5.E-01	0.051	4.E-03	0.025	4.E-06						
17	rs8072199	26116848	A	0.074	5.E-07	0.067	2.E-04	0.019	7.E-01						
19	**rs283815**	45390333	G	-0.006	7.E-01	-0.067	3.E-03	-0.031	4.E-06	-0.032	2.E-06	-0.022	4.E-03	-0.002	2.E-01
19	rs71352238	45394336	G	-0.035	1.E-01	-0.106	4.E-05	-0.043	7.E-08	-0.025	2.E-03	-0.016	8.E-02	-0.002	2.E-01
19	rs2075650	45395619	G	-0.032	1.E-01	-0.104	6.E-05	-0.043	**5.E-08**	-0.029	3.E-04	-0.019	3.E-02	-0.002	2.E-01
19	**rs157582**	45396219	A	-0.011	5.E-01	-0.074	8.E-04	-0.033	1.E-06	-0.032	2.E-06	-0.021	5.E-03	-0.002	2.E-01
19	rs769449	45410002	A	-0.052	2.E-02	-0.123	1.E-05	-0.045	1.E-07	-0.029	1.E-03	-0.019	5.E-02	0.000	8.E-01
20	rs11906369	46829233	A	-0.123	2.E-06	-0.129	5.E-05	-0.007	5.E-01						
20	rs6512614	48792663	C	-0.002	9.E-01	-0.047	1.E-02	-0.028	5.E-07						
20	rs6012871	48792992	G	-0.001	1.E+00	-0.046	1.E-02	-0.028	6.E-07						
20	rs6125931	48795413	G	-0.001	1.E+00	-0.047	9.E-03	-0.028	4.E-07						
20	rs6125934	48803937	A	0.005	7.E-01	-0.046	1.E-02	-0.030	6.E-08						
20	rs6025261	55503259	A	0.079	9.E-08	0.082	5.E-06	0.005	4.E-01						

Notes.
Genome build GRCh37.
Blue (bold) SNP names indicates the SNPs are listed for suggested association with more than one phenotype at the p<5E-06 level in the HRS discovery cohort.
Orange (bold) effect sizes and p-values indicates the SNP association surpassed the genome-wide significance threshold of 5E-08 in the HRS discovery cohort.
Green shading indicates the association exceeds 5E-06 threshold in the HRS discovery cohort.
Pink shading indicates that the association with other phenotypes are provided for comparison.
Brown shading indicates that the results for the marker are for a correlated phenotype, with results provided for comparison.

Table D. Top hit SNPs from GWAS on rDR-L in the ELSA sample, that were not found in HRS

				Minor		ELSA		HRS	
Chr.	SNP	Position	Ref. allele	allele (freq.)	Gene(s)	Beta	P	Beta	P
16	rs4783859	54997905	C	C (0.43)	--	-0.034	4.E-06	0.031	6.E-01
16	rs12931755	54998943	C	G (0.17)	--	-0.048	9.E-10	0.040	5.E-01
16	rs4258610	55000195	G	T (0.17)	--	-0.058	1.E-10	0.036	6.E-01
16	rs28413114	55002902	T	T (0.42)	--	-0.060	4.E-11	0.032	6.E-01
16	rs34851234	55003947	T	T (0.14)	--	-0.047	3.E-09	0.056	3.E-01
16	rs35826089	55012475	T	A (0.14)	--	-0.052	1.E-08	-0.017	8.E-01
16	rs34473486	55013607	A	T (0.14)	--	-0.057	3.E-10	0.032	6.E-01
16	rs35755352	55017281	T	A (0.16)	--	-0.047	7.E-08	0.053	4.E-01
16	rs4436774	55018268	A	A (0.15)	--	-0.045	1.E-07	0.062	3.E-01
16	rs4784445	55020068	A	T (0.16)	--	-0.049	2.E-08	0.060	4.E-01
16	rs4784446	55020841	T	A (0.16)	--	-0.045	1.E-07	0.066	3.E-01
16	rs12448424	55020849	A	T (0.15)	--	-0.045	2.E-07	0.068	3.E-01
16	rs12448424	55021482	T	T (0.15)	--	-0.045	1.E-07	0.066	3.E-01

Notes.
Genome build GRCh37.
-- indicates there was no known gene location, but all SNPs listed were flanked by *LOC100132339* to the left, and *IRX6* to the right
