# Supplementary material for: Genetic variants specific to aging-related verbal memory: Insights from GWASs in a population-based cohort
Source: PLoS One. 2017 Aug 11;12(8):e0182448. doi: 10.1371/journal.pone.0182448 (PMC5553750; doi:10.1371/journal.pone.0182448)
Supplement: S2 File — (PDF) [file pone.0182448.s009.pdf]

Meta-Analysis Results

Tables below provide top SNPs associated with each phenotype at the suggestive level of a statistical significance, $5x10^{-06}$ for immediate recall level (IR-L; Table A), immediate recall change (IR-C; Table B), residual delayed recall level (rDR-L; Table C), and residual delayed recall change (rDR-C; Table D).

Table A. Top SNP associations with IR-L from the meta-analysis of HRS and ELSA GWAS results

Chr	SNP Name	Location	Effect Allele	Weight	Zscore	P-value	Direction
13	rs1449587	49413468	A	13930	-4.863	1.16E-06	--
3	rs4122385	188140653	A	14013	-4.746	2.08E-06	--
3	rs2162259	188161706	A	14017	-4.73	2.25E-06	--
8	rs79129295	50225832	A	13954	4.594	4.35E-06	++

Table B. Top SNP associations with IR-C from the meta-analysis of HRS and ELSA GWAS results

Chr	SNP Name	Location	Effect Allele	Weight	Zscore	P-value	Direction
19	rs157582	45396219	A	13163	-6.139	8.29E-10	--
19	rs283815	45390333	G	13032	-6.008	1.88E-09	--
19	rs769449	45410002	A	13179	-5.593	2.24E-08	--
19	rs2075650	45395619	G	13198	-5.324	1.01E-07	--
17	rs8073765	13629731	G	13195	-4.88	1.06E-06	--
19	rs71352238	45394336	G	13169	-4.859	1.18E-06	--
8	rs73526541	1074467	A	13207	-4.66	3.16E-06	--
6	rs760608	114719447	G	13196	-4.632	3.63E-06	--

Table C. Top SNP associations with rDR-L from the meta-analysis of HRS and ELSA GWAS results

Chr	SNP Name	Location	Effect Allele	Weight	Zscore	P-value	Direction
19	rs769449	45410002	A	13992	-6.975	3.05E-12	--
19	rs2075650	45395619	G	14011	-6.57	5.03E-11	--
19	rs71352238	45394336	G	13981	-6.46	1.04E-10	--
19	rs157582	45396219	A	13972	-5.791	7.01E-09	--
19	rs283815	45390333	G	13842	-5.403	6.55E-08	--
4	rs705119	72613036	A	14006	4.921	8.60E-07	++
1	rs6571943	9132520	A	14013	4.865	1.14E-06	++
7	rs16873450	23806640	A	14013	4.857	1.19E-06	++
4	rs705120	72614140	A	14005	4.856	1.20E-06	++
14	rs1950326	40039316	T	14007	4.801	1.58E-06	++
4	rs7041	72618334	A	14012	4.759	1.95E-06	++
14	rs4902774	40032181	G	13974	4.723	2.33E-06	++
19	rs34095326	45395844	A	13993	-4.685	2.80E-06	--
7	rs13240694	23564901	A	13926	4.682	2.84E-06	++
6	rs9380152	11234035	A	14012	4.676	2.93E-06	++
7	rs7792388	23541266	A	14019	4.675	2.94E-06	++
7	rs4722238	23568653	G	14012	4.662	3.13E-06	++
1	rs12117043	9132520	A	13999	4.624	3.76E-06	++
1	rs875995	9098272	A	13938	4.618	3.88E-06	++
20	rs6123551	54762418	G	14001	4.609	4.04E-06	++
4	rs222040	72616932	G	14012	4.607	4.09E-06	++
4	rs222047	72610208	A	13992	4.601	4.21E-06	++

Table D. Top SNP associations with rDR-C from the meta-analysis of HRS and ELSA GWAS results

Chr	SNP Name	Location	Effect Allele	Weight	Zscore	P-value	Direction
8	rs2974279	88075043	A	13066	-4.74	2.14E-06	--
17	rs72823319	37280517	A	13156	-4.634	3.59E-06	--
17	rs7225562	37267827	G	12995	-4.625	3.74E-06	--
7	rs10949834	73480569	A	13076	-4.62	3.84E-06	--
8	rs4596672	88055465	G	13147	-4.587	4.50E-06	--
