# Supplementary material for: Genetic variants specific to aging-related verbal memory: Insights from GWASs in a population-based cohort
Source: PLoS One. 2017 Aug 11;12(8):e0182448. doi: 10.1371/journal.pone.0182448 (PMC5553750; doi:10.1371/journal.pone.0182448)
Supplement: S3 File — (PDF) [file pone.0182448.s010.pdf]

Additional Evaluation Using the Inferred *APOE* Genotypes

APOE imputation.

For chromosome 19, there were 42,471 directly genotyped SNPs used to impute 469,034 total SNPs to the 1000 Genomes reference panel [1, 2]. The two SNPs used to determine the *APOE* e2/e3/e4 isoform status were among those imputed (rs7412 and rs429358, imputed with certainties of .998 and .997, respectively). Thus, we did not include *APOE* genotypes in our initial analysis. Yet, because of the estimated accuracy of imputation methods used [3-9], and particularly for *APOE* isoform status [10, 11], we used the *APOE* genotype to conduct sensitivity analyses to further evaluate the validity of our main findings. With regard to *APOE* imputation in particular, prior validation has been conducted when using similar pre-phasing and imputation approaches as with HRS. Imputed *APOE* SNPs have demonstrated a high degree of agreement with directly genotyped SNPs (kappa coefficients were between 0.92-0.94 for rs429358, and 0.90-0.93 for rs7412 depending upon study sample [11]). In the HRS sample, the inferred genotype frequencies (e2/e2 at 0.6%, e2/e3 at 12.6%, e2/e4 at 2.3%, e3/e3 at 60.7%, e3/e4 at 21.8%, and e4/e4 at 2.0%) were in Hardy-Weinberg equilibrium ($X^2(2)=0.68$) and were similar to those in another community-based longitudinal study (e2/e2 at 0.4%, e2/e3 at 13.7%, e2/e4 at 1.3%, e3/e3 at 63.9%, e3/e4 at 19.0%, and e4/e4 at 1.7%; [12]).

It should be noted that from the discovery GWAS in HRS, our top SNP association with rDR-L in the *TOMM40* region (rs2075650) was used as one of the 42,471 imputation basis SNPs. It is unclear how this influences the joint effect of rs2075650 and *APOE* genotype although, the pairwise LD between rs2075650 and rs7412 is $r^2=0.014$, and with rs429358 is $r^2=0.565$, calculated using Phase 3 of the 1000 Genomes Project, for the CEU panel [13]. Additional analyses performed indicate that both *TOMM40* and *APOE* retain overlapping and

independent effects on rDR level. Similarly, as shown in results in the main paper, there were significant effects of both the *TOMM40* SNP (rs2075650) and the top *APOE* SNP (rs769449), both directly genotyped on rDR-L.

The top SNP association from the meta-analysis of IR-C in *TOMM40* (rs157582) was also an imputation basis SNP. The pairwise LD between rs157582 and rs7412 is r^2=0.008, and with rs429358 is r^2=0.590 [13]. Additional analysis performed on the effect of *TOMM40* rs157782 and *APOE* genotypes is reported in the main paper, with the effects showing some overlapping and independent effects.

Sensitivity Testing.

The effects of *TOMM40* SNPs on both rDR-L and IR-C differed by *APOE* genotype, shown in Tables S8-S9. For rDR-L, effect sizes for the G allele were strongest in individuals with e2/e3 and e2/e4 genotypes, although with non-significant p-values that could be due to the lower number of individuals with these genotypes. For IR-C, although there were significant effects of the rs157582 A allele within the e3/e3 and e2/e3 strata, the effect size was strongest among individuals with the e2/e4 genotype, albeit not significant.

Table A. Results from testing the effects of *TOMM40* (rs2075650) 'G' allele on rDR level within each *APOE* genotype strata

Genotype	b	SE	p-value	Model sample size
e2/e2	nr[a]			N=43
e2/e3	-0.0539	0.0544	.322	N=942
e2/e4	-0.0648	0.0525	.219	N=171
e3/e3	-0.0390	0.0180	.031	N=4545
e3/e4	-0.0177	0.0187	.345	N=1631
e4/e4	0.0102	0.0485	.834	N=150

[a] nr = Not run because none of the e2/e2 individuals carry the G allele

Table B. Results from testing the effects of *the TOMM40* (rs157582) 'A' allele on IR change within each *APOE* genotype strata

Genotype	b	SE	p-value	Model sample size
e2/e2	0.0816	0.1414	.568	N=37
e2/e3	-0.0477	0.0221	.032	N=846
e2/e4	-0.1221	0.1245	.328	N=153
e3/e3	-0.0310	0.0155	.047	N=4043
e3/e4	-0.0106	0.0366	.772	N=1433
e4/e4	nr[a]			N=149

[a] nr = Not run because all of the e4/e4 individuals carry the A allele

Size of genetic effects

Findings from multiple sources [14, 15] implicate polygenic influences on components of verbal memory, so individual alleles are not expected to have large effect sizes. The largest effect was for rs150510877 with IR-L, which had an effect size of .18 SD per risk allele. On average, a 60-year old person homozygous for the risk allele is predicted to have an immediate recall score that is 0.32 words lower (2 alleles x 0.1586) than a 60-year old with zero risk alleles, or the same score as a 66-year old with no risk alleles (based on the expected decline of .05 words per year, Table 2).

Our analyses find that the included SNPs account for only a portion of the genetic effect on components of verbal memory, with the heritability in DR is mostly due to IR. The cumulative polygenic effect may be additive (from positive and negative effects of 'risk' alleles [16]), multiplicative with gene-by-gene interactions (i.e., GxG [17]), or a combination. While pathway analysis depicts that there is evidence of direct and indirect GxG effects on memory phenotypes involving *TOMM40* in AD-related processes, distinguishing these processes requires very large samples with repeated assessments and will require further investigation through future studies.

Testing of Potential Confounders

Because we did not include potential confounders (e.g., smoking, BMI, educational attainment, socioeconomic resources), as a sensitivity check for our top hit, we ran a linear regression model for the effect of the *TOMM40* SNP on rDR level, adjusting for sex, *APOE* e4 carrier status, 3 PCs, and educational attainment. The effect of the *TOMM40* remained (b=-0.0251, SE=0.0108, p=.020, r2=0.0136) with there being smaller effects from education (b=0.0118, SE=0.0015, p<.0001). As another check, we ran a phenotypic regression to test the additional effects of smoking, BMI, and household income on rDR level to estimate how much of the variance these variables account for; the variance accounted for was very small (r2=0.0155). This suggests that even if there was a completely causal relationship between these factors and rDR level, they would account for very little of the variation in it.

References

1. CIDR. CIDR Health and Retirement Study: Imputation Report - 1000 Genomes Project reference panel. Seattle, WA: University of Washington, 2012.
2. Faul J, Smith J, Zhao W. Health and Retirement Study: Candidate Genes for Cognition/Behavior. Ann Arbor, MI: University of Michigan, 2014.
3. Hao K, Chudin E, McElwee J, Schadt EE. Accuracy of genome-wide imputation of untyped markers and impacts on statistical power for association studies. BMC genetics. 2009;10(1):1.
4. Howie B, Fuchsberger C, Stephens M, Marchini J, Abecasis GR. Fast and accurate genotype imputation in genome-wide association studies through pre-phasing. Nat Genet. 2012;44(8):955-9.
5. Howie BN, Donnelly P, Marchini J. A flexible and accurate genotype imputation method for the next generation of genome-wide association studies. PLoS Genet. 2009;5(6):e1000529. Epub 2009/06/23. doi: 10.1371/journal.pgen.1000529. PubMed PMID: 19543373; PubMed Central PMCID: PMC2689936.
6. Li Y, Willer C, Sanna S, Abecasis G. Genotype imputation. Annu Rev Genomics Hum Genet. 2009;10: :387-406. Epub 2009/09/01. doi: 10.1146/annurev.genom.9.081307.164242. PubMed PMID: 19715440; PubMed Central PMCID: PMC2925172.
7. Marchini J, Howie B, Myers S, McVean G, Donnelly P. A new multipoint method for genome-wide association studies by imputation of genotypes. Nat Genet. 2007;39(7):906-13.
8. Nho K, Shen L, Kim S, Swaminathan S, Risacher SL, Saykin AJ, et al., editors. The effect of reference panels and software tools on genotype imputation. AMIA Annual Symposium Proceedings; 2011: American Medical Informatics Association.
9. Porcu E, Sanna S, Fuchsberger C, Fritsche LG. Genotype imputation in Genome‐Wide association studies. Current protocols in human genetics. 2013:1.25. 1-1.. 14.
10. Oldmeadow C, Holliday EG, McEvoy M, Scott R, Kwok JB, Mather K, et al. Concordance between direct and imputed APOE genotypes using 1000 Genomes data. Journal of Alzheimer's Disease. 2014;42(2):391-3.
11. Radmanesh F, Devan WJ, Anderson CD, Rosand J, Falcone GJ. Accuracy of imputation to infer unobserved APOE epsilon alleles in genome-wide genotyping data. European Journal of Human Genetics. 2014;22(10):1239-42.
12. Lahoz C, Schaefer EJ, Cupples LA, Wilson PW, Levy D, Osgood D, et al. Apolipoprotein E genotype and cardiovascular disease in the Framingham Heart Study. Atherosclerosis. 2001;154(3):529-37.
13. Machiela MJ, Chanock SJ. LDlink: a web-based application for exploring population-specific haplotype structure and linking correlated alleles of possible functional variants. Bioinformatics. 2015;31(21):3555-7.
14. Davies G, Tenesa A, Payton A, Yang J, Harris SE, Liewald D, et al. Genome-wide association studies establish that human intelligence is highly heritable and polygenic. Molecular psychiatry. 2011;16(10):996-1005.

15. Kremen WS, Panizzon MS, Franz CE, Spoon KM, Vuoksimaa E, Jacobson KC, et al. Genetic complexity of episodic memory: A twin approach to studies of aging. Psychol Aging. 2014;29(2):404.
16. Dudbridge F. Power and predictive accuracy of polygenic risk scores. PLoS Genet. 2013;9(3):e1003348. Epub 2013/04/05. doi: 10.1371/journal.pgen.1003348. PubMed PMID: 23555274; PubMed Central PMCID: PMC3605113.
17. Thomas D. Gene--environment-wide association studies: emerging approaches. Nat Rev Genet. 2010;11(4):259-72. Epub 2010/03/10. doi: 10.1038/nrg2764. PubMed PMID: 20212493; PubMed Central PMCID: PMC2891422.
